# Associations of cumulative exposure and dynamic trajectories of the combined atherogenic-frailty index with incident cardiometabolic multimorbidity: a prospective cohort study

**DOI:** 10.7189/jogh.16.04319

**Published:** 2026-10-19

**Authors:** Haidi Wu, Zikai Song, Tengfei Ye, Chunhua Yang

**Affiliations:** 1 Department of Cardiology, The First Hospital of Jilin University, Changchun, China; 2 Department of Geriatrics, Ruijin Hospital Lu Wan Branch, Shanghai Jiaotong University School of Medicine, Shanghai, China

**Keywords:** atherogenic index of plasma-frailty index, cardiometabolic multimorbidity, cumulative exposure, K-means clustering, CHARLS

## Abstract

**Background:**

Cardiometabolic multimorbidity (CMM) poses a growing global health burden, yet effective tools for early risk stratification remain limited. The combined impact of atherogenic dyslipidaemia and physiological frailty, captured by the atherogenic index of plasma-frailty index (AIP-FI), on CMM development is poorly understood. This study aimed to investigate the associations of cumulative exposure and longitudinal trajectories of AIP-FI with incident CMM.

**Methods:**

This prospective cohort study included 4,157 participants aged ≥45 years without baseline CMM from CHARLS. The AIP-FI was constructed as the product of AIP and a 37-deficit frailty index. Cumulative AIP-FI was calculated from repeated measurements. Longitudinal trajectories were identified using K-means clustering. Associations with incident CMM were evaluated by multivariable Cox models, RCS, K-M curves, ROC/NRI/IDI, mediation, interaction, subgroup, and sensitivity analyses.

**Results:**

Over a mean 8.5-year follow-up, 612 incident CMM cases occurred. Both higher baseline and cumulative AIP-FI were significantly associated with increased CMM risk, with cumulative exposure showing a stronger effect (fully-adjusted HR per SD increment: 1.46; 95% CI = 1.38–1.54). Participants in the highest quartile of cumulative AIP-FI had a near 4-fold higher CMM risk compared to the lowest (HR = 3.95; 95% CI = 2.95–5.29). Clustering analyses revealed that groups with high-stable AIP-FI exhibited the highest CMM risks. Cumulative AIP-FI demonstrated superior predictive performance for CMM (AUC = 71.17%) and significantly improved risk reclassification over traditional markers (NRI = 0.157). Mediation analyses suggested bidirectional synergistic effects between AIP and FI.

**Conclusions:**

In this national cohort, both the cumulative burden and adverse longitudinal trajectories of AIP-FI were independently associated with an elevated risk of CMM. Dynamic monitoring of this composite biomarker could facilitate the early identification of high-risk individuals, thereby informing targeted prevention strategies for middle-aged and older adults in China.

Cardiometabolic multimorbidity (CMM), defined as the concurrent diagnosis of ≥2 conditions among heart disease, stroke and diabetes mellitus (DM) [1,2], poses a substantial global health challenge. Recent epidemiological studies indicate that approximately 18.5% of middle-aged and older adults worldwide are affected by CMM, with prevalence increasing by 38.7% over the past three decades due to population aging and obesogenic lifestyles [3,4]. This condition markedly elevates all-cause mortality risk, disability rates, and healthcare utilisation, substantially increasing hospitalisation needs and per-patient costs (averaging €12,400 annually) [5–7]. Despite its profound burden, early identification of high-risk individuals remains challenging due to the absence of validated integrative biomarkers. The atherogenic index of plasma (AIP) is a robust biomarker of atherogenic lipoprotein heterogeneity and insulin resistance [8]. It demonstrates significant associations with hypertension, DM, and metabolic dysregulation, and independently predicts incident stroke, heart disease, and major adverse cardiovascular events [9–13]. Frailty index, a validated metric of biological aging, quantifies multisystem decline *via* deficit accumulation and independently predicts cardiovascular risks, stroke, and all-cause mortality [14–17]. However, current evidence is predominantly from single-domain assessments.

The dynamic and cumulative synergy between metabolic dysfunction and physiological vulnerability, particularly in driving CMM progression, remains insufficiently investigated, representing a critical gap in risk-stratification. We therefore developed the AIP-FI composite to quantify this dual burden. Existing evidence indicates that AIP-FI, integrating metabolic and biological-aging dimensions, is a promising cardiovascular risk tool [18]. Biologically, these two domains are not merely additive but may amplify each other through shared inflammatory, metabolic, and vascular mechanisms [19,20]. Therefore, a multiplicative index better captures their synergy than an additive measure. Using the nationally representative China Health and Retirement Longitudinal Study (CHARLS) cohort, we examined the association between AIP-FI and CMM, emphasising cumulative exposure burden and longitudinal trajectories, to establish dynamic AIP-FI monitoring as an early predictor of CMM and inform prevention strategies.

## METHODS

### Study design and populations

This study used data from the CHARLS, a national prospective cohort launched in 2011 with a stratified, multistage, probability-proportional-to-size design covering 28 provinces, 150 counties, and 450 communities. The baseline survey recruited 17,708 adults aged ≥45 years (plus a small number of younger spouses) and was followed in 2013, 2015, 2018, and 2020 through face-to-face, computer-assisted personal interviews conducted every 2–3 years. Blood was collected in waves 1 and 3 to support the cumulative-change analyses. After excluding participants with age <45 years, missing baseline measurements, prevalent CMM, or incomplete follow-up, the final analytic cohort included 4,157 participants (**Figure 1**). We conducted our analysis in accordance with the Journal of Global Health’s GRABDRO [21] (Table S1 in the **Online Supplementary Document**). Baseline characteristics of included and excluded participants showed that the included cohort was representative, with minor differences likely due to the large sample size (Table S2 in the **Online Supplementary Document**).

**Figure 1:**
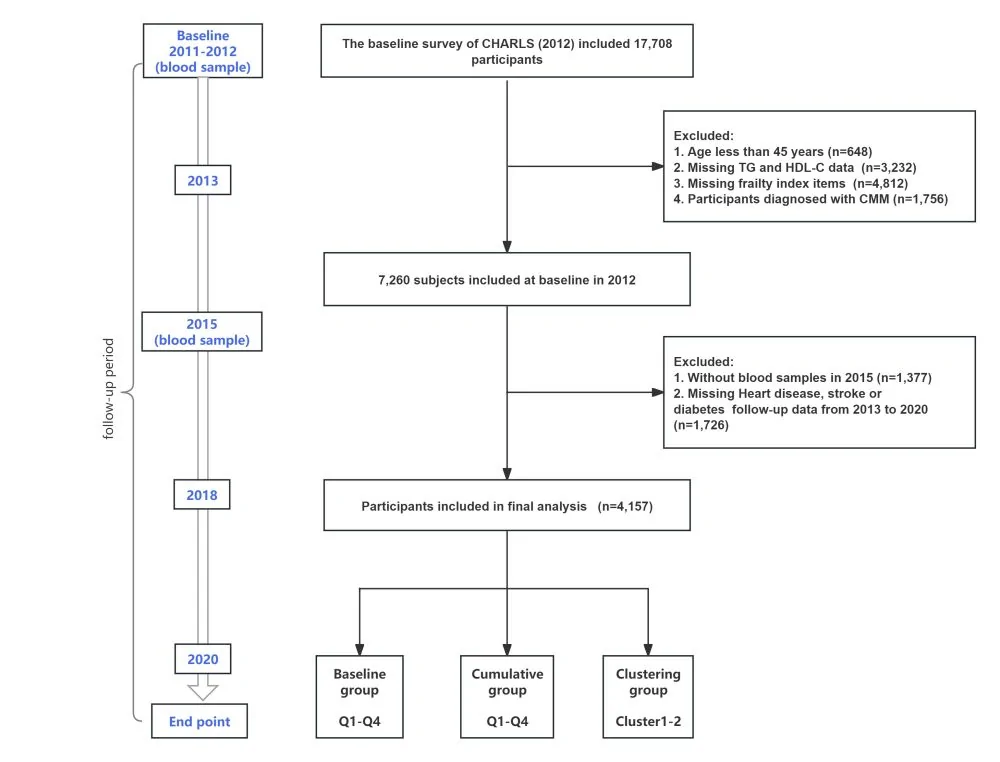
Flowchart of study participants.

#### Assessment of AIP-FI and its changes

AIP was calculated as the log-transformed ratio of triglycerides to high-density lipoprotein cholesterol (HDL-C) [22]. Cumulative AIP (cumAIP) was derived as the time-weighted average of the AI*P* values measured in 2012 and 2015, reflecting the average exposure over the interval. Frailty was assessed using the Rockwood cumulative-deficit approach, incorporating 37 deficits across activities of daily living, instrumental activities, physical function, chronic conditions (excluding heart disease, stroke, and DM), cognitive function, and self-rated health [18,23,24] (Table S3 in the **Online Supplementary Document**). Individual FI was defined as the sum of present deficits divided by 37. The cumulative frailty index (cumFI) was calculated as the time-weighted average of the FI values measured in 2012, 2013, and 2015. To quantify combined exposure, we derived multiplicative indices, including the baseline AIP-FI and the cumulative AIP-FI, by multiplying the respective baseline or cumulative values of AIP and FI [25]. Relative to simple averaging, spline-based area-under-the-curve, or mixed-effects approaches, this metric accounts for exposure duration, is robust to irregular measurements, yields individual-level values suitable for risk classification, and is clinically simple to compute [26–29]. K-means clustering of 2012–2015 AIP-FI measurements identified two clusters (low: 81.5%, high: 18.5%) with k = 2 by elbow method (Figure S1, Panels A–C in the **Online Supplementary Document**). Given only two time points, K-means was preferred over trajectory models for efficiency and interpretability. The formulas for the above-mentioned indices are as follows:

*AIP = log_10_ (TG (mg/dL) / HDL-C (mg/dL))*

*cumAIP = (AIP_2012_ + AIP_2015_) / 2 × (2015–2012)*

*cumFI = (FI_2012_ + FI_2013_ + FI_2015_) / 3 × (2015–2012)*

*AIP – FI = AIP × FI*

*cumAIP – FI = cumAIP × cumFI*

#### Assessment of CMM and individual CMDs

The primary outcome was CMM, defined as the co-occurrence of two or more conditions among heart disease, stroke, and DM. Secondary outcomes were the incident events of these cardiovascular metabolic diseases (CMDs) individually [30,31]. Heart disease and stroke were ascertained from self-reported physician diagnoses or documented medication use. Diabetes mellitus was defined by self-reported physician diagnosis, current glucose-lowering or insulin therapy, fasting plasma glucose (FPG)≥126 mg/dL, or glycated haemoglobin (HbA1c) ≥ 6.5% [32]. Outcomes were derived from face-to-face interviews, medication inventories, and laboratory assessments, and event timing was set at the first diagnosis or measurement meeting the criteria [33,34].

### Covariates

Baseline (Wave 1) data were used. Standardised questionnaires captured demographics (age, sex, residence, marital status, education), lifestyle (smoking, drinking, sleep duration), physical activity, and histories of hypertension and dyslipidaemia. Sleep duration was recorded biennially. Standardised measurements included height, weight, and waist circumference; body mass index (BMI) was calculated as weight in kilograms divided by the square of height in meters, and blood pressure was measured to calculate mean systolic blood pressure (SBP) and diastolic blood pressure (DBP). Fasting blood from waves 1 and 3 was analysed at the College of American Pathologists-accredited You'anmen Clinical Laboratory Center for FPG, total cholesterol (TC), triglycerides (TG), HDL-C, low-density lipoprotein cholesterol (LDL-C), HbA1c, and uric acid (UA). Hypertension was defined as self-reported diagnosis, current antihypertensive use, systolic pressure ≥140 mm Hg, or diastolic pressure ≥90 mm Hg [35]. Dyslipidemia by TG≥150, TC≥240, HDL-C<40, or LDL-C≥160 mg/dL, self-reported diagnosis, or current lipid-lowering use [36]. The triglyceride-glucose (TyG) index was derived from the natural logarithm of the product of fasting plasma glucose and triglycerides divided by two [37], and estimated glucose disposal rate (eGDR) derived using the Williams formula [38].

### Statistical analyses

Continuous variables are presented as mean and standard deviation or median, categorical variables as frequencies (%), with groups compared using appropriate tests. Using serial (2012, 2015) AIP-FI measurements, K-means clustering identified two groups (k = 2 by the elbow method). Cox proportional-hazards models assessed the associations of continuous, quartile-based, and cluster forms of baseline and cumulative AIP-FI with incident CMM and individual CMDs. To minimise over-adjustment, models were incrementally adjusted: Model 1 for age and sex; Model 2 additionally for demographic factors; Model 3 further for metabolic and clinical variables (sleep duration, physical activity, BMI, hypertension, dyslipidaemia, LDL-C, uric acid) that may lie on the causal pathway, with the progressive attenuation of hazard ratios across models reflecting these intermediates. The proportional-hazards (PH) assumption was tested with Schoenfeld residuals, missing data were handled by multiple imputation by chained equations (MICE) based on 20 data sets (Table S4 in the **Online Supplementary Document**), and multicollinearity was assessed with variance inflation factors (VIFs). The results are presented in Table S5 in the **Online Supplementary Document**. Nonlinear relationships were explored with restricted cubic splines (3 knots), cumulative incidence with Kaplan-Meier curves, and predictive performance compared by receiver operating characteristic (ROC) analysis with net reclassification improvement (NRI) and integrated discrimination improvement (IDI). Causal mediation analysis and tests of multiplicative and additive interaction, quantifying the relative excess risk due to interaction (RERI), attributable proportion (AP), and synergy index (SI), were also performed. Subgroup analyses were stratified by age, sex, residence, education, smoking, drinking, and BMI. Sensitivity analyses comprised age-stratified, logistic-regression, complete-case, and E-value approaches.

All statistical analyses were conducted using *R* Statistical software, version 4.2.2 (The R Foundation, Vienna, Austria) and Free Statistics Analysis Platform, version 2.4.0 (Beijing, China). A two-sided *P*-value <0.05 was considered statistically significant.

## RESULTS

### Baseline characteristics

Baseline characteristics of the 4,157 participants are shown by incident CMM status (**Table 1**). Those who developed CMM (n = 612, 14.7%) were older, more often female, and had shorter sleep and lower physical activity. They also had more adverse anthropometric, metabolic, and lipid profiles-higher BMI, waist circumference, blood pressure, FBG, HbA1c, TC, TG, LDL-C, and TyG index, with lower HDL-C and eGDR (all *P* < 0.001), and more prevalent hypertension, dyslipidaemia, heart disease, stroke, and DM (all *P* < 0.001). All atherogenicity and frailty indices and their clusters were correspondingly worse in this group (all *P* < 0.01). A consistent gradient emerged across quartiles, cumulative exposure, and cluster groupings, with higher AIP-FI levels tracking deteriorating metabolic profiles and rising prevalence of CMM and its components (Tables S6–8 in the **Online Supplementary Document**).

**Table 1:** Descriptive characteristics of the participants at baseline

Characteristic	Total	CMM	Non-CMM	*P*-value
	(n = 4,157)	(n = 612)	(n = 3,545)	
Age in years (x̄ ± SD)	60.0 ± 8.7	60.7 ± 8.1	59.9 ± 8.8	0.032
Sex, n (%)				0.012
*Female*	2,486 (59.8)	394 (64.4)	2,092 (59)	
*Male*	1,671 (40.2)	218 (35.6)	1,453 (41)	
Residence, n (%)				0.258
*Urben*	1,331 (32.0)	208 (34)	1,123 (31.7)	
*Rural*	2,826 (68.0)	404 (66)	2,422 (68.3)	
Educational level, n (%)				0.42
*No formal education*	2,225 (53.6)	316 (51.8)	1,909 (53.9)	
*Elementary school*	936 (22.5)	141 (23.1)	795 (22.4)	
*Middle and high school*	707 (17.0)	102 (16.7)	605 (17.1)	
*Vocational school and above*	286 (6.9)	51 (8.4)	235 (6.6)	
Marital status, n (%)				0.123
*Married*	3,632 (87.4)	523 (85.5)	3,109 (87.7)	
*Others*	525 (12.6)	89 (14.5)	436 (12.3)	
Smoking status, n (%)				0.001
*Yes*	1,132 (27.2)	134 (21.9)	998 (28.2)	
*No*	3,025 (72.8)	478 (78.1)	2,547 (71.8)	
Drinking status, n (%)				0.021
*Yes*	1,201 (28.9)	153 (25)	1,048 (29.6)	
*No*	2,956 (71.1)	459 (75)	2,497 (70.4)	
Sleep duration in hours (x̄ ± SD)	6.2 ± 1.9	5.9 ± 1.9	6.2 ± 1.9	<0.001
Physical activity, n (%)				<0.001
*Low*	604 (34.1)	119 (43)	485 (32.5)	
*Moderate and high*	1,167 (65.9)	158 (57)	1,009 (67.5)	
BMI,kg/m^2^, Mdn (IQR)	23.2 (20.9, 26.1)	25.3 (22.3, 27.9)	23.0 (20.7, 25.6)	<0.001
Waist circumference in cm (x̄ ± SD)	84.7 ± 12.6	89.3 ± 13.2	83.9 ± 12.3	<0.001
SBP in mmHg (x̄ ± SD)	129.4 ± 21.7	134.9 ± 21.6	128.5 ± 21.5	<0.001
DBP in mmHg (x̄ ± SD)	75.1 ± 12.2	77.8 ± 12.0	74.6 ± 12.2	<0.001
FBG in mg/dL (x̄ ± SD)	108.9 ± 34.0	121.2 ± 51.9	106.7 ± 29.4	<0.001
TC in mg/dL (x̄ ± SD)	194.0 ± 38.1	201.6 ± 37.9	192.7 ± 38.0	<0.001
TG in mg/dL, Mdn (IQR)	106.2 (76.1, 154.9)	124.8 (88.5, 183.4)	103.5 (74.3, 149.6)	<0.001
HDL-C in mg/dL (x̄ ± SD)	51.2 ± 15.4	47.7 ± 15.0	51.9 ± 15.4	<0.001
LDL-C in mg/dL (x̄ ± SD)	116.7 ± 34.8	123.6 ± 37.2	115.5 ± 34.2	<0.001
HbAlc (x̄ ± SD)	5.3 ± 0.8	5.5 ± 1.1	5.2 ± 0.7	<0.001
UA in mg/dL (x̄ ± SD)	4.3 ± 1.2	4.5 ± 1.3	4.3 ± 1.2	0.015
TyG (x̄ ± SD)	8.7 ± 0.7	8.9 ± 0.7	8.6 ± 0.6	<0.001
eGDR (x̄ ± SD)	9.3 ± 2.0	8.9 ± 2.1	9.3 ± 2.0	<0.001
Hypertension, n (%)	1,990 (47.9)	413 (67.5)	1,577 (44.5)	<0.001
Dyslipedaemia, n (%)	453 (10.9)	150 (24.5)	303 (8.5)	<0.001
Heart disease, n (%)	1,398 (33.6)	536 (87.6)	862 (24.3)	<0.001
Stroke, n (%)	534 (12.8)	304 (49.7)	230 (6.5)	<0.001
DM, n (%)	1,244 (29.9)	491 (80.2)	753 (21.2)	<0.001
AIP, Mdn (IQR))	0.3 (0.1, 0.6)	0.4 (0.2, 0.7)	0.3 (0.1, 0.5)	<0.001
FI (x̄ ± SD)	0.1 ± 0.1	0.2 ± 0.1	0.1 ± 0.1	<0.001
cumAIP, Mdn (IQR)	1.1 (0.5, 1.6)	1.3 (0.8, 1.9)	1.0 (0.5, 1.6)	<0.001
cumFI, x̄ ± SD	0.9 ± 0.4	1.1 ± 0.4	0.8 ± 0.4	<0.001
AIP-FI, SD, Mdn (IQR)	−0.3 (−0.6, 0.4)	0.3 (−0.4, 1.2)	−0.3 (−0.7, 0.2)	<0.001
cumAIP-FI, SD, Mdn (IQR)	−0.2 (−0.7, 0.6)	0.4 (−0.1, 1.1)	−0.3 (−0.8, 0.4)	<0.001
AIP-FI K-means group, n (%)				<0.001
*Cluster1*	3,386 (81.5)	375 (61.3)	3,011 (84.9)	
*Cluster2*	771 (18.5)	237 (38.7)	534 (15.1)	

AIP – the atherogenic index of plasma, BMI – body mass index, CMM – cardiometabolic multimorbidity, cumAIP – cumulative atherogenic index of plasma, cumFI – cumulative frailty index, DBP – diastolic blood pressure, DM – diabetes mellitus, eGDR – estimated glucose disposal rate, FBG – fasting blood-glucose, FI – frailty index, HbA1c – glycosylated hemoglobin A1c, HDL-C – high-density lipoprotein cholesterol, IQR – interquartile range, LDL-C – low-density lipoprotein cholesterol, SBP – systolic blood pressure, SD – standard deviation, TC – total cholesterol, TG – triglyceride, UA – uric acid, TyG – triglyceride-glucose, x̄ – mean

### Associations of baseline and longitudinal AIP-FI trajectories with CMM

During a mean follow-up of 8.5 years, higher baseline and cumulative AIP-FI were significantly associated with increased CMM risk, with stronger associations for cumulative exposure (**Table 2**). In the fully adjusted model, each 1-standard deviation (SD) increase in baseline AIP-FI was associated with a 31% higher risk (hazard ratio (HR) = 1.31; 95% confidence interval (CI) = 1.23–1.39), and each 1-SD increment in cumulative AIP-FI with a 46% higher risk (HR = 1.46; 95% CI = 1.38–1.54). Quartile analyses showed a graded relationship for both, with highest-*vs.*-lowest HRs of 3.95 (95% CI = 2.95–5.29) and 2.40 (95% CI = 1.86–3.09), respectively. The high-stable cluster likewise carried a higher CMM risk than the low-stable cluster (HR = 2.06; 95% CI = 1.72–2.46). Parallel associations were observed for heart disease, stroke, and DM (Table S9 in the **Online Supplementary Document**).

**Table 2:** Association of baseline and longitudinal AIP-FI trajectories with the incidence of CMM

Variable	n.event %	Model 1*		Model 2*		Model 3*	
		HR (95% CI)	*P*-value	HR (95% CI)	*P*-value	HR (95% CI)	*P*-value
AIP-FI (per 1 SD)	612 (14.7)	1.42 (1.35 ~ 1.49)	<0.001	1.43 (1.36 ~ 1.5)	<0.001	1.31 (1.23 ~ 1.39)	<0.001
AIP-FI quartile							
*Q1*	86 (8.3)	1(Ref)		1(Ref)		1(Ref)	
*Q2*	105 (10.1)	1.2 (0.9 ~ 1.59)	0.217	1.18 (0.89 ~ 1.57)	0.257	1.11 (0.83 ~ 1.48)	0.474
*Q3*	135 (13)	1.64 (1.25 ~ 2.15)	<0.001	1.63 (1.24 ~ 2.13)	<0.001	1.33 (1.01 ~ 1.75)	0.044
*Q4*	286 (27.5)	3.47 (2.73 ~ 4.42)	<0.001	3.46 (2.71 ~ 4.41)	<0.001	2.4 (1.86 ~ 3.09)	<0.001
cumAIP-FI (per 1 SD)	612 (14.7)	1.54 (1.47 ~ 1.61)	<0.001	1.56 (1.49 ~ 1.63)	<0.001	1.46 (1.38 ~ 1.54)	<0.001
cumAIP-FI quartile							
*Q1*	59 (5.7)	1(Ref)		1(Ref)		1(Ref)	
*Q2*	90 (8.7)	1.55 (1.12 ~ 2.16)	0.009	1.55 (1.11 ~ 2.15)	0.009	1.38 (0.99 ~ 1.92)	0.056
*Q3*	149 (14.3)	2.66 (1.97 ~ 3.6)	<0.001	2.68 (1.98 ~ 3.62)	<0.001	2.13 (1.57 ~ 2.9)	<0.001
*Q4*	314 (30.2)	5.7 (4.31 ~ 7.54)	<0.001	5.73 (4.32 ~ 7.58)	<0.001	3.95 (2.95 ~ 5.29)	<0.001
K-means group							
*Cluster1*	375 (11.1)	1(Ref)		1(Ref)		1(Ref)	
*Cluster2*	237 (30.7)	2.87 (2.43 ~ 3.38)	<0.001	2.69 (2.28 ~ 3.18)	<0.001	2.06 (1.72 ~ 2.46)	<0.001

AIP – the atherogenic index of plasma, CI – confidence interval, CMM – cardiometabolic multimorbidity, cumAIP-FI – cumulative atherogenic index of plasma-frailty index, FI – frailty index, HR – hazard ratio, SD – standard deviation, Ref – reference

*Model 1 adjusted for age and sex; Model 2 adjusted for variables in Model 1 and residence, educational level, marital status, smoking status, and drinking status; Model 3 adjusted for variables in Model 2 and sleep duration, physical activity, body mass index, history of hypertension and dyslipidaemia, LDL-C and UA.

Restricted cubic spline analyses revealed a nonlinear dose-response relationship for baseline AIP-FI, with increasing hazard ratios across the exposure spectrum (**Figure 2**, Panel A). Similarly, cumulative AIP-FI demonstrated the same nonlinear pattern (**Figure 2**, Panel B). Associations with heart disease, stroke, and DM were positive and linear, from baseline to cumulative forms (Figure S2, Panels A–F in the **Online Supplementary Document**).

**Figure 2:**
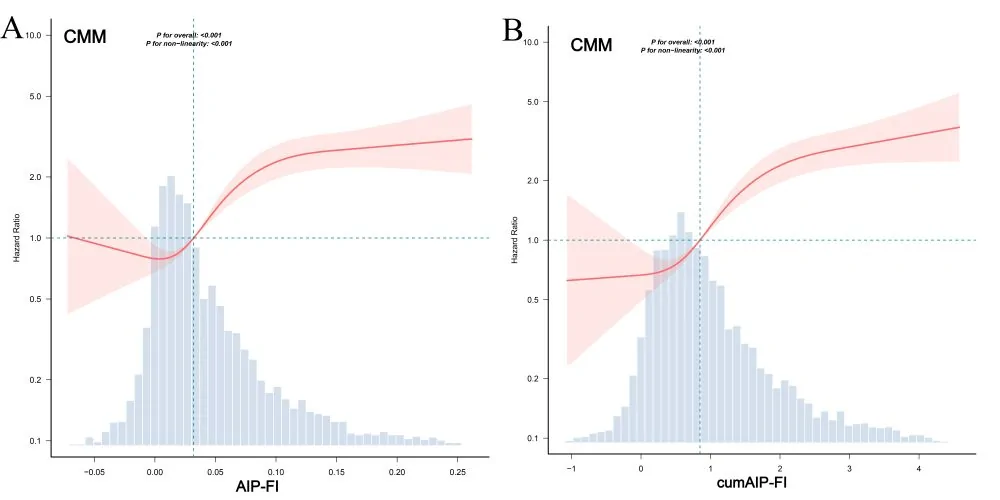
Association of AIP-FI and cumulative AIP-FI with CMM risk after covariate adjustment. Panel A. Restricted cubic spline curve for CMM by AIP-FI. Panel B. Restricted cubic spline curve for CMM by cumulative AIP-FI. Heavy central line represents the estimated adjusted hazard ratio, with shaded ribbons denoting 99.8% confidence interval. The model is adjusted for age, sex, residence, educational level, marital status, smoking status, and drinking status, sleep duration, physical activity, body mass index, history of hypertension and dyslipidaemia, LDL-C and UA. AIP – the atherogenic index of plasma, CMM – cardiometabolic multimorbidity, cumAIP-FI – cumulative atherogenic index of plasma-frailty index, DM – diabetes mellitus, FI – frailty index.

Kaplan-Meier analyses showed significantly higher cumulative incidence across all AIP-FI groupings for every outcome (log-rank *P* < 0.05 for all). For CMM, cumulative incidence increased progressively with higher cumulative AIP-FI quartiles and in the high-stable cluster, with less pronounced differences for baseline quartiles (Figure S3, Panels A–C in the **Online Supplementary Document**). Consistent patterns were seen for heart disease, stroke, and DM (Figure S4, Panels A–I in the **Online Supplementary Document**).

### Predictive performance and incremental value

Cumulative AIP-FI showed the best predictive performance for CMM (AUC = 71.17%), followed by baseline AIP-FI (66.26%) and other indices ranged from 57.12% to 61.33% (**Figure 3**). Adding cumulative AIP-FI to the basic model significantly improved reclassification and discrimination (NRI = 0.157, IDI = 0.024), as shown in Table S10 in the **Online Supplementary Document**. CumAIP (NRI = 0.118) and TyG (NRI = 0.093) showed modest NRI improvements without significant IDI, while eGDR showed significant IDI improvement (IDI = 0.011) without NRI improvement. Adding baseline AIP-FI did not significantly improve either metric (Table S11 in the **Online Supplementary Document**).

**Figure 3:**
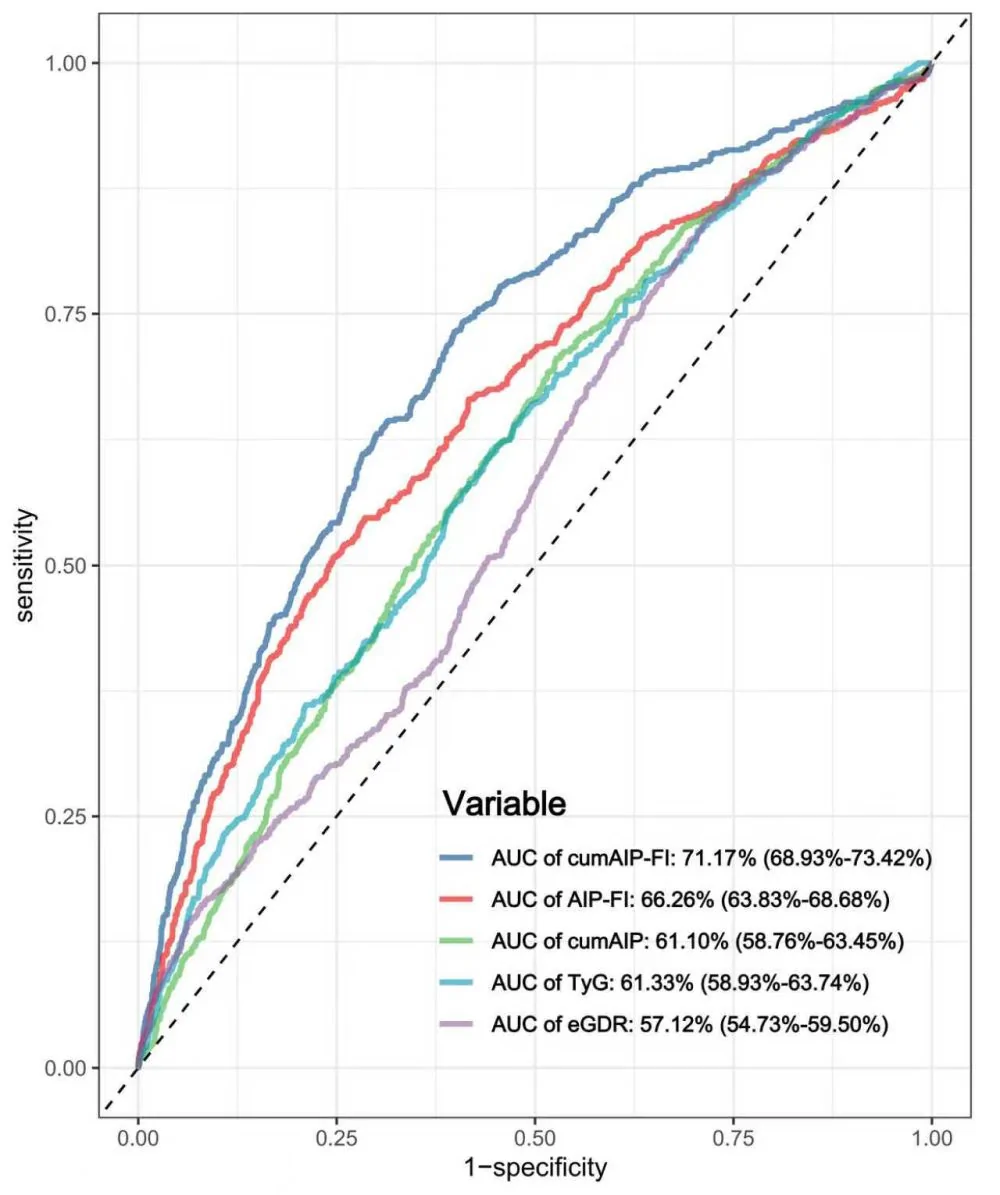
Receiver operating characteristic curve analysis of the AIP-FI in predicting the risk of CMM. AUC – area under the curve, AIP – the atherogenic index of plasma, cumAIP – cumulative atherogenic index of plasma, cumAIP-FI – cumulative atherogenic index of plasma-frailty index, eGDR – estimated glucose disposal rate, FI – frailty index, TyG – triglyceride-glucose.

### Mediation and interaction effects

Mediation analyses showed that FI significantly mediated the association between AIP and CMM (15.95%, indirect = 0.7%, total = 4.39%, *P* < 0.001), and AIP significantly mediated the association between FI and CMM (3.54%, indirect = 0.38%, total = 10.81%, *P* < 0.001). The corresponding mediation models are presented (Figure S5, Panels A and B in the **Online Supplementary Document**). For components, FI mediated AIP effects on heart disease (55.22%), stroke (19.05%), and DM (4.88%); AIP mediated FI effects on stroke (2.76%) and DM (10.48%), but not heart disease (Figure S6, Panels A–F in the **Online Supplementary Document**).

Interaction analysis showed significant additive interaction between cumulative AIP and FI on CMM (RERI = 0.96, AP = 0.20, SI = 1.34), also for stroke and DM, but not heart disease; no multiplicative interaction was found (**Table 3**).

**Table 3:** Impact of cumulative AIP and FI interaction on CMM, heart disease, stroke, and DM

Interactive items*	Crude model		Fully adjusted model†	
	Coefficients (95% CI)	*P*-value	Coefficients (95% CI)	*P*-value
**CMM**				
Additive effect				
*RERI*	2.4 (0.89, 3.9)	<0.001	0.96 (0.14, 1.78)	0.01
*AP*	0.25 (0.12, 0.39)	<0.001	0.2 (0.04, 0.37)	0.01
*SI*	1.39(1.13, 1.73)	<0.001	1.34 (1.01, 1.79)	<0.001
Multiplicative effect	0.71 (0.46, 1.1)	0.21	0.95 (0.64, 1.43)	0.82
**Heart disease**				
Additive effect				
*RERI*	0.25 (-0.11, 0.6)	0.08	0.11 (−0.23, 0.45)	0.26
*AP*	0.1 (−0.04, 0.23)	0.08	0.05(−0.1, 0.91)	0.26
*SI*	1.19 (0.91, 1.54)	<0.001	1.09 (0.82, 1.45)	0.03
Multiplicative effect	1 (0.8, 1.25)	0.98	0.99 (0.79, 1.24)	0.92
**Stroke**				
Additive effect				
*RERI*	0.89 (0.05, 1.72)	0.02	0.69 (0.1, 1.48)	0.04
*AP*	0.19 (0.02, 0.37)	0.02	0.17 (−0.02, 0.35)	0.04
*SI*	1.33 (0.98, 1.78)	<0.001	1.28 (0.93, 1.75)	<0.001
Multiplicative effect	0.98 (0.65, 1.46)	0.92	0.97 (0.65, 1.45)	0.87
**DM**				
Additive effect				
*RERI*	0.57 (0.19, 0.94)	<0.001	0.39 (0.03, 0.74)	0.02
*AP*	0.2 (0.07, 0.33)	<0.001	0.15 (0.01, 0.29)	0.02
*SI*	1.44(1.08, 1.91)	<0.001	1.35(0.98, 1.86)	<0.001
Multiplicative effect	1.06(0.83, 1.34)	0.64	1.04 (0.82, 1.31)	0.76

AIP – the atherogenic index of plasma, AP – attribute proportion, CMM – cardiometabolic multimorbidity, CI – confidence interval, DM – diabetes mellitus, FI – frailty index, RERI – relative excess risk due to interaction, SI – synergy index

*In the absence of interaction RERI = 0, AP = 0, and SI = 1.

†Fully model adjusted for age, sex, residence, educational level, marital status, smoking status, and drinking status, sleep duration, physical activity, body mass index, history of hypertension and dyslipidaemia, low-density lipoprotein cholesterol, and uric acid.

### Subgroup and sensitivity analyses

Subgroup analyses were conducted to assess the consistency of the positive association between cumAIP-FI and CMM risk. The association remained significant across all strata defined by age, sex, residence, education level, smoking status, drinking status, or BMI, with no significant interaction observed (all P for interaction >0.05) (Figure S7 in the **Online Supplementary Document**). Sensitivity analyses confirmed the robustness of the findings. Age-stratified analysis revealed consistent dose–response relationships in both younger (HR = 5.53) and older adults (HR = 3.19). Additionally, logistic regression yielded odds ratios consistent with Cox model results. Third, complete-case analysis showed no meaningful change in effect estimates after excluding participants with missing data. Fourthly, E-value analysis indicated that unmeasured confounding was unlikely to fully explain the observed association (E-value = 2.28). Finally, Schoenfeld residual tests confirmed that the proportional hazards assumption was satisfied for the primary exposure variables across all Cox models (all *P* > 0.05 for the exposure terms) (Tables S12–16 in the **Online Supplementary Document**).

## DISCUSSION

This study establishes that the AIP-FI, a composite biomarker of metabolic-physiological frailty, effectively predicts CMM risk in middle-aged and older Chinese adults. Both cumulative exposure and dynamic trajectories of AIP-FI were independently associated with CMM and individual CMDs, persisting after multivariable adjustment. A nonlinear dose-response relationship was observed. CumAIP-FI demonstrated superior discriminative ability and improved risk reclassification over conventional biomarkers. Interaction and mediation analyses revealed a bidirectional amplification between atherogenic dyslipidaemia and physiological reserve depletion. These findings suggest that dynamic AIP-FI monitoring may aid in early risk stratification and could potentially inform targeted prevention strategies, though further interventional studies are needed to establish clinical utility.

Atherogenic dyslipidaemia and physiological system frailty, have well-established research foundations in the fields of cardiovascular and metabolic diseases, respectively. Numerous studies have demonstrated that higher baseline AIP levels are significantly associated with an increased risk of coronary heart disease, stroke, and metabolic multimorbidity [9,39–42]. Additionally, AIP has been identified as an important predictor of diabetes onset and progression [12,43,44]. The FI, a composite measure of declining physiological reserve across multiple systems, has been shown to effectively predict the incidence of various age-related diseases, mortality, and the coexistence of CMM [18,45–49]. Individuals with higher FI values typically exhibit elevated inflammatory levels, metabolic disturbances, and organ dysfunction. As a composite integrating these two dimensions, AIP-FI holds potential for CMM prediction, yet its use has been little studied. To our knowledge, only Huo *et al*. have explored baseline AIP-FI and cardiovascular events, and their work considered single-time point levels only [18]. While the cumulative effects and trajectories of individual biomarkers have been examined [10,50–52], none has addressed these dynamic aspects of the composite AIP-FI. Our study is the first to investigate the associations of cumulative AIP-FI burden and its longitudinal trajectories with incident CMM and each CMD in a large prospective cohort.

Earlier work examined AIP-FI and cardiovascular events [18] and the dynamics of AIP alone with CMM [53], but neither addressed the combined effect of cumulative AIP-FI burden and its trajectories on CMM. Higher baseline AIP-FI values were associated with increased CMM risk, and cumulative exposure, along with trajectory clustering, identified the high-stable group as having significantly higher risk than the low-stable group. Notably, we first demonstrate a nonlinear dose-response relationship between cumulative AIP-FI and CMM, with better predictive performance than traditional biomarkers. Dynamic monitoring captures risk evolution and may facilitate early intervention, while maintaining a low-stable trajectory could provide a critical preventive window. Nevertheless, the predictive gains were modest but statistically significant, reclassifying about one-sixth of participants. We propose AIP-FI as a complementary screening tool for:

1. dynamic risk monitoring;

2. identifying high-risk phenotypes;

3. providing a low-cost, accessible index for resource-limited settings.

It may enhance population-level screening but should be combined with established risk factors for individual decisions. Future interventional studies are needed to confirm its clinical utility. Moreover, the mediating effect of FI on the AIP and outcome associations varied markedly: strongest for heart disease, intermediate for stroke, and weakest for diabetes. This gradient may reflect pathophysiological differences, as heart disease, particularly coronary artery disease, is driven by atherosclerotic plaque formation, a process intimately linked to vascular aging and frailty [54,55]. The high mediation proportion suggests that much of the AIP-heart disease association may operate through frailty-related pathways, including chronic inflammation, endothelial dysfunction, and reduced cardiovascular reserve. This is consistent with recent Mendelian randomisation evidence demonstrating a bidirectional causal relationship between frailty and coronary atherosclerosis [56]. Stroke encompasses heterogeneous aetiologies – including atherothrombotic, cardioembolic, and lacunar subtypes – with only a subset directly linked to atherosclerosis [57]. This aetiological heterogeneity may explain the moderate mediation proportion, as frailty-related pathways may be more relevant for large-artery atherosclerotic stroke than for other subtypes. Previous studies have reported that frailty is associated with increased stroke risk, with hypertension and hyperlipidaemia mediating approximately 23.6% and 14.2% of this association, respectively [58]. Diabetes development involves predominantly metabolic mechanisms, including insulin resistance and β-cell dysfunction, that may be more directly influenced by atherogenic dyslipidaemia itself rather than through frailty pathways [59]. The low mediation proportion suggests that AIP exerts a relatively direct metabolic effect on diabetes risk, largely independent of global physiological decline, consistent with evidence linking metabolic syndrome components to frailty [60]. Additionally, prior studies have shown frailty to be more strongly associated with cardiovascular than with metabolic outcomes in older adults [34,61]. The attenuation of hazard ratios after full adjustment supports partial mediation through metabolic pathways, implying that fully adjusted estimates may underrepresent the total effect of AIP-FI on CMM.

The underlying mechanisms linking AIP-FI to CMM remain unclear but likely involve a complex network of interactions. Metabolically, atherogenic dyslipidaemia may impair vascular and metabolic function through lipotoxic cascades, in which retention of triglyceride-rich lipoproteins beneath the endothelium triggers endoplasmic-reticulum stress, insulin resistance, and disrupted glucose homeostasis [62,63]. Concurrently, HDL dysfunction may impair cholesterol efflux and ApoA-I binding to pancreatic beta-cells, diminishing glucose-stimulated insulin secretion [64]. Oxidative stress could further amplify this damage, as AIP-mediated lipid peroxidation promotes reactive oxygen species (ROS) and vascular adhesion molecules, potentially accelerating atherosclerotic plaque formation [41,65]. From a physiological-aging perspective, frailty and aging may drive immune dysregulation and low-grade chronic inflammation. The persistent release of pro-inflammatory cytokines, such as IL-6 and TNF-alpha, has been hypothesised to promote insulin resistance and destabilise atherosclerotic plaques [62,66]. Notably, we observed a bidirectional association between AIP and FI, with FI showing a stronger mediating effect, and one plausible pathway is that atherosclerosis may increase CMM risk partly by exacerbating frailty. Interaction analyses revealed significant positive synergy between AIP and FI for CMM and its components. Mechanistically, elevated inflammatory cytokines during atherosclerosis may not only accelerate vascular lesions but also impair muscle, nerve, and skeletal function, exacerbating frailty; chronic inflammation in a frail state may in turn accelerate atherosclerosis, forming a vicious cycle [67,68]. Reduced cardiovascular reserve and diminished stress adaptation in frail individuals may further increase cardiometabolic risk [66,69]. This bidirectional interaction, if confirmed, could contribute substantially to CMM risk.

Our study has several strengths. We are the first to apply AIP-FI to CMM risk prediction, establishing a metabolism-physiology dual-system framework beyond traditional single indicators. Four-dimensional analyses of baseline, cumulative, clustered, and trajectory perspectives revealed spatiotemporal risk patterns. We confirmed a dose-response effect of cumulative AIP-FI and demonstrated the independent predictive value of dynamic trajectories, supporting their role in monitoring high-risk phenotypes and guiding intervention. Additionally, we found bidirectional mediation and synergistic amplification between AIP and FI, providing empirical support for the lipid metabolism-frailty vicious cycle theory. Despite these strengths, several limitations should be acknowledged. First, the CHARLS cohort comprised middle-aged and older Chinese adults, limiting generalisability to younger populations and other ethnic groups. Second, residual confounding may persist despite multivariable adjustment. Third, heart disease and stroke were self-reported, which is subject to recall bias and may miss undiagnosed cases. Fourth, the mediation analyses were based on observational data and the assumption of no unmeasured confounding; thus, the mediation proportions should be considered exploratory rather than causal. Fifth, clustering relied on only two time points and cannot capture nonlinear trajectory patterns, highlighting the need for future studies with more frequent measurements.

## CONCLUSIONS

Drawing on evidence from a nationwide prospective cohort, both the cumulative burden and dynamic trajectories of AIP-FI exhibited independent and robust positive associations with the risk of CMM and its subtypes. Cumulative AIP-FI demonstrated the optimal predictive capability for CMM and significantly improved the risk stratification performance of traditional models. Atherogenic dyslipidaemia and physiological system vulnerability appear to be associated with CMM development, possibly through a bidirectional synergistic amplification effect. Dynamic monitoring of AIP-FI may serve as a tool for early identification of high-risk CMM phenotypes, offering a new rationale for optimising CMM risk stratification and developing targeted prevention strategies in clinical practice.

## Additional material


Online Supplementary Document

## Data Availability

**Data availability:** Data set are distributable only by CHARLS team and are available in public domain through registration on CHARLS website: http://charls.pku.edu.cn.
