# Supplementary material for: Associations of cumulative exposure and dynamic trajectories of the combined atherogenic-frailty index with incident cardiometabolic multimorbidity: a prospective cohort study
Source: J Glob Health. 2026 Oct 19;16:04319. doi: 10.7189/jogh.16.04319 (PMC13679350; doi:10.7189/jogh.16.04319)
Supplement: Supplementary file 1 [file jogh-16-04319-s001.pdf]

Supplement to: Haidi W, Zikai S, Tengfei Y, Chunhua Y. Associations of cumulative exposure and dynamic trajectories of the combined atherogenic-frailty index with incident cardiometabolic multimorbidity: a prospective cohort study. J Glob Health. 2026;16:04319.

Legend:

Table S1 Adherence to JoGH’s GRABDROP guidelines items
Table S2 Baseline characteristics of included versus excluded participants aged ⩾45 years.
Table S3 List of health deficits items included in the frailty index.
Table S4 Distribution of variables with missing data.
Table S5 Collinearity statistics.
Table S6 Descriptive characteristics of the participants at baseline AIP-FI.
Table S7 Descriptive characteristics of the participants at cumulative AIP-FI.
Table S8 Descriptive characteristics of the participants at clustering of AIP-FI.
Table S9 Association of baseline and longitudinal AIP-FI trajectories with the incidence of heart disease, stroke and DM
Table S10 Predictive value of AIP-FI for incident CMM.
Table S11 Incremental Predictive Value of Baseline and Cumulative AIP-FI for CMM.
Table S12 Cox regression for the association of baseline and cumulative AIP-FI with the incidence of CMM by age group.
Table S13 Logical analysis for the association between baseline and cumulative AIP-FI with the incidence of CMM.
Table S14 Cox regression analysis of the association between baseline and cumulative AIP-FI with the incidence of CMM after excluding participants with missing values (n=846).
Table S15 E-values for the association between AIP-FI and new-onset incident CMM.
Table S16 Schoenfeld Residuals Test for Proportional Hazards Assumption.

Figure S1 Clustering of the change in the AIP-FI from 2012-2015
Figure S2 Restricted cubic spline curves for CMDs after covariate adjustment.
Figure S3 Kaplan-Meier curves of cumulative incidence of CMM based on baseline, cumulative, longitudinal clustering AIP-FI groupings.
Figure S4 Kaplan-Meier curves of cumulative incidence of heart disease, stroke, and DM based on baseline, cumulative, longitudinal clustering AIP-FI groupings.
Figure S5 Mediation analysis of FI and AIP on the risk of CMM.
Figure S6 Mediation analysis of FI and AIP on the risk of CMDs.
Figure S7 Subgroup analysis of the association between cumulative AIP-FI and CMM

Table S1 Adherence to JoGH's GRABDROP guidelines items

JoGH guideline items

1. Please list all papers published by each co-author in previous 3 years that were based on secondary analysis of a big data repository

Haidi Wu. Association of serum uric acid to all-cause and cardiovascular mortality in patients with cardiovascular disease. Sci Rep. 2024 Sep 18;14(1):21808. doi: 10.1038/s41598-024-72527-4.

Zikai Song. Association of serum uric acid to all-cause and cardiovascular mortality in patients with cardiovascular disease. Sci Rep. 2024 Sep 18;14(1):21808. doi: 10.1038/s41598-024-72527-4.

Chunhua Yang. Association between sarcopenia and falls in Chinese older adults: Findings from the China health and retirement longitudinal study. PLoS One. 2025 Jun 12;20(6):e0326193. doi: 10.1371/journal.pone.0326193.

2. Please explain the key elements of your study design and the use of the available datasets that make your study an original scientific contribution

First, this study separately computed cumulative AIP (cumAIP, derived from two repeated measurements in 2012 and 2015) and cumulative FI (cumFI, integrating three time points: 2012, 2013, and 2015), then derived the multiplicative composite index (cumAIP-FI = cumAIP × cumFI). This approach more accurately characterizes the independent longitudinal trajectory of each component before assessing their joint effect, representing a methodologically more rigorous quantification of cumulative dual-system burden.

Second, to our knowledge, this is the first study to conduct bidirectional mediation analyses between AIP and FI to disentangle their independent and indirect contributions to CMM risk. The results revealed that FI significantly mediated 15.95% of the association between AIP and CMM, while AIP significantly mediated 3.54% of the association between FI and CMM. Furthermore, this is also the first study to simultaneously evaluate both additive (RERI, AP, SI) and multiplicative interactions between cumulative AIP and cumulative FI, demonstrating a significant synergistic effect on CMM incidence (RERI = 0.96, AP = 0.20, SI = 1.34). These analyses provide novel mechanistic evidence supporting the "lipid metabolism–physiological frailty" vicious cycle.

Third, cumulative AIP-FI achieved an AUC of 71.17% for predicting CMM, with significant improvement in risk reclassification over traditional biomarkers (NRI = 0.157, IDI = 0.024), indicating superior predictive and discriminative capacity.

3. Please list all publications that addressed similar research questions in the same dataset and indicate where you cited them in your paper

Huo et al. (2025). Association between combined atherogenic and frailty index and cardiovascular events: evidence from the CHARLS cohort. Cardiovasc Diabetol 25:33. This article is discussed in the third paragraph of the Introduction, where it is cited as Reference [18].

Hu & Zhao (2026). Longitudinal cohort study on the dynamic changes of atherogenic index of plasma and the risk of cardiometabolic multimorbidity. BMC Cardiovasc Disord. This article is discussed in the second paragraph of the Discussion, where it is cited as Reference [18] and [54].

4. Please explain how you addressed multiple testing through an appropriately rigorous statistical threshold and indicate this in the methods section

In our original manuscript, we clearly distinguished between the primary outcome (CMM)

and secondary outcomes (heart disease, stroke, and DM). All primary analyses focused on CMM as the main exposure-outcome relationship, while analyses of individual cardiometabolic diseases were treated as exploratory secondary outcomes. This a priori distinction reduces the burden of multiple comparisons for the primary research question.

We employed three incrementally adjusted models (Model 1–3) to demonstrate the consistency of associations across confounding adjustment levels, rather than conducting multiple independent hypothesis tests. This approach provides internal validation of result stability rather than inflating the Type I error rate.

The consistency of dose-response relationships across quartile-based analyses, continuous analyses, and restricted cubic spline curves provides corroborative evidence that reduces the likelihood that significant findings are attributable to multiple testing artifacts.

5. Please declare to what extent have AI chatbots been used in developing your paper and to which parts of the paper did they contribute

The authors declare that no AI chatbots or large language models were used in the development of this manuscript. All writing, analyses, and interpretations were conducted entirely by the listed authors.

Table S2 Baseline characteristics of included versus excluded participants aged ≥45 years

Characteristic	Total (n = 17060)	Included (n = 4157)	Excluded (n = 12903)	p
Age,years(Mean±SD)	59.4 ± 9.8	60.0 ± 8.7	59.2 ± 10.1	< 0.001
Sex,n(%)				< 0.001
Female	8738 (51.2)	2486 (59.8)	6252 (48.5)	
Male	8322 (48.8)	1671 (40.2)	6651 (51.5)	
Residence,n(%)				< 0.001
Urben	6907 (40.5)	1331 (32)	5576 (43.2)	
Rural	10153 (59.5)	2826 (68)	7327 (56.8)	
Educational level,n(%)				< 0.001
No formal education	7733 (45.5)	2225 (53.6)	5508 (42.8)	
Elementary school	3573 (21.0)	936 (22.5)	2637 (20.5)	
Middle and High school	3536 (20.8)	707 (17)	2829 (22)	
Vocational school and above	2171 (12.8)	286 (6.9)	1885 (14.7)	
Marital status,n(%)				0.426
Married	14823 (87.0)	3632 (87.4)	11191 (86.9)	
Others	2213 (13.0)	525 (12.6)	1688 (13.1)	
Smoking status,n(%)				< 0.001
Yes	4788 (29.4)	1132 (27.2)	3656 (30.1)	
No	11501 (70.6)	3025 (72.8)	8476 (69.9)	
Drinking status,n(%)				< 0.001
Yes	5631 (33.3)	1201 (28.9)	4430 (34.7)	
No	11284 (66.7)	2956 (71.1)	8328 (65.3)	
Sleep duration,hours,(Mean ± SD)	6.4 ± 1.9	6.2 ± 1.9	6.4 ± 1.9	< 0.001
Physical activity (%)				0.029
Low	2416 (36.2)	604 (34.1)	1812 (37)	
Moderate and High	4250 (63.8)	1167 (65.9)	3083 (63)	
BMI,kg/m2,(Median(IQR))	23.1 (20.8, 25.7)	23.2 (20.9, 26.1)	23.1 (20.7, 25.6)	0.003
Waist circumference(cm), Mean ± SD	84.3 ± 12.6	84.7 ± 12.6	84.1 ± 12.6	0.016
SBP,mmHg(Mean ± SD)	129.8 ± 21.5	129.4 ± 21.7	130.0 ± 21.5	0.175
DBP,mmHg(Mean ± SD)	75.5 ± 12.2	75.1 ± 12.2	75.7 ± 12.2	0.004
FBG,mg/dL(Mean ± SD)	110.4 ± 37.3	108.9 ± 34.0	111.4 ± 39.1	< 0.001
TC,mg/dL(Mean ± SD)	193.3 ± 38.8	194.0 ± 38.1	192.9 ± 39.2	0.15
TG,mg/dL(Median (IQR))	106.2 (75.2, 156.6)	106.2 (76.1, 154.9)	106.2 (75.2, 157.5)	0.997
HDL-C,mg/dL(Mean ± SD)	50.9 ± 15.4	51.2 ± 15.4	50.7 ± 15.3	0.099
LDL-C,mg/dL(Mean ± SD)	116.3 ± 34.9	116.7 ± 34.8	116.0 ± 35.0	0.31
HbAlc,%(Mean ± SD)	5.3 ± 0.8	5.3 ± 0.8	5.3 ± 0.9	0.123
UA,mg/dL(Mean ± SD)	4.5 ± 1.3	4.3 ± 1.2	4.6 ± 1.3	< 0.001
tyg.11, Mean ± SD	8.7 ± 0.7	8.7 ± 0.7	8.7 ± 0.7	0.165
eGDR.11, Mean ± SD	9.1 ± 2.1	9.3 ± 2.0	9.0 ± 2.1	< 0.001
hibpe_1, n (%)	7479 (44.3)	1990 (47.9)	5489 (43.1)	< 0.001
dyslipe, n (%)	1656 (10.0)	453 (10.9)	1203 (9.7)	0.023
allHeart.01, n (%)	4678 (27.4)	1398 (33.6)	3280 (25.4)	< 0.001
allStroke.01, n (%)	1809 (10.6)	534 (12.8)	1275 (9.9)	< 0.001
allDM.01, n (%)	4189 (24.6)	1244 (29.9)	2945 (22.8)	< 0.001
allCMM.01, n (%)	2142 (12.6)	612 (14.7)	1530 (11.9)	< 0.001

AIP.11, Median (IQR)	0.3 (0.1, 0.6)	0.3 (0.1, 0.6)	0.3 (0.1, 0.6)	0.409
FI2011, Mean ± SD	0.1 ± 0.1	0.1 ± 0.1	0.2 ± 0.1	< 0.001
cumAIP, Median (IQR)	1.1 (0.5, 1.6)	1.1 (0.5, 1.6)	1.1 (0.5, 1.7)	0.198
cumFI, Mean ± SD	0.5 ± 0.3	0.5 ± 0.2	0.5 ± 0.3	< 0.001
AIP-FI,SD (Median (IQR))	-0.3 (-0.6, 0.3)	-0.3 (-0.6, 0.2)	-0.2 (-0.6, 0.5)	< 0.001
cumAIP-FI,SD,(Median (IQR))	-0.2 (-0.7, 0.4)	-0.3 (-0.7, 0.3)	0.8 (0.1, 1.9)	< 0.001

BMI - body mass index, SBP - systolic blood pressure, DBP - diastolic blood pressure, FBG - Fasting blood-glucose, TC - total cholesterol, TG - triglyceride, HDL-C - high-density lipoprotein cholesterol, LDL-C - low-density lipoprotein cholesterol, HbA1c - glycosylated hemoglobin A1c, UA - uric acid, TyG - triglyceride-glucose, eGDR - estimated glucose disposal rate, DM - diabetes Mellitus, CMM - cardiometabolic multimorbidity, AIP - the atherogenic index of plasma, F - frailty index, cumAIP - cumulative atherogenic index of plasma, cumFI - cumulative frailty index, IQR - interquartile range, SD - standard deviation

Table S3 List of health deficits items included in the frailty index.

Type of deficit	Item	Variables	Cut-off point
Activities of daily living	1	Bathing	No=0; a little =0.33; need help = 0.67; Yes=1
	2	Dressing	No=0; a little =0.33; need help = 0.67; Yes=1
	3	Use of toilet	No=0; a little =0.33; need help = 0.67; Yes=1
	4	Transferring	No=0; a little =0.33; need help = 0.67; Yes=1
	5	Continence	No=0; a little =0.33; need help = 0.67; Yes=1
	6	Eating	No=0; a little =0.33; need help = 0.67; Yes=1
Instrumental activities of daily living	7	Cooking	No=0; a little =0.33; need help = 0.67; Yes=1
	8	Shopping	No=0; a little =0.33; need help = 0.67; Yes=1
	9	Doing household	No=0; a little =0.33; need help = 0.67; Yes=1
	10	Taking medicine	No=0; a little =0.33; need help = 0.67; Yes=1
	11	Managing money	No=0; a little =0.33; need help = 0.67; Yes=1
Physical functional limitations	12	Lift a weight of 5 kg	No=0; a little =0.33; need help = 0.67; Yes=1
	13	Walking 1 km	No=0; a little =0.33; need help = 0.67; Yes=1
	14	Stooping, kneeling, or crouching	No=0; a little =0.33; need help = 0.67; Yes=1
	15	Able to stand up from sitting	No=0; a little =0.33; need help = 0.67; Yes=1
	16	Able to pickup a coin from a table	No=0; a little =0.33; need help = 0.67; Yes=1
	17	Running or jogging about 1 km	No=0; a little =0.33; need help = 0.67; Yes=1
	18	Reaching or extend arms	No=0; a little =0.33; need help = 0.67; Yes=1
	19	Climbing several flights of stairs without resting	No=0; a little =0.33; need help = 0.67; Yes=1
Chronic disease (Excluding such as heart, stroke and diabetes)	20	Psych problem.	Yes=1, no=0
	21	Arthritis.	Yes=1, no=0
	22	Liver disease.	Yes=1, no=0
	23	Kidney disease.	Yes=1, no=0
	24	Stomach/digestive disease.	Yes=1, no=0
	25	Chronic lung diseases	Yes=1, no=0
	26	Memory problem.	Yes=1, no=0
Disability	27	Physical disabilities	Yes=1, no=0
	28	Brain damage/mental retardation	Yes=1, no=0
	29	Vision problem	Yes=1, no=0
	30	Hearing problem	Yes=1, no=0
	31	Speech impediment	Yes=1, no=0
Mental health	32	Feel (no) hopeful about the future	Always=1; often=0.67; sometimes=0.33; seldom or never=0
	33	Feel fearful	Always=1; often=0.67; sometimes=0.33; seldom or never=0
	34	Feel everything was an effort	Always=1; often=0.67; sometimes=0.33; seldom or never=0

	35	Feel could not get "going"	Always=1; often=0.67; sometimes=0.33; seldom or never=0
	36	Sleep was restless	Always=1; often=0.67; sometimes=0.33; seldom or never=0
Subjective functioning	37	Self-rated health	Very good=0; good=0.25; average=0.5; bad=0.75; very bad=1

Table S4 Distribution of variables with missing data.

Variables	Number of missing	Proportion of missing
BMI	391	9.41%
Educational level	3	0.07%
Physical activity	2386	57.40%
Sleep duration	20	0.48%
LDL	8	0.19%
UA	1	0.02%
eGDR	401	9.65%
TyG	3	0.07%
FI_2013	1289	31%
FI_2015	1269	30.53%

BMI - body mass index, LDL-C - low-density lipoprotein cholesterol, UA - uric acid, eGDR - estimated glucose disposal rate, TyG - triglyceride-glucose, FI - frailty index

Table S5 Collinearity statistics.

Variable	GVIF	DF	GVIF^(1/(2*Df))
Crude	1.235	1.0	1.111
Age	1.376	1.0	1.173
Sex	1.908	1.0	1.381
Residence	1.131	1.0	1.063
Educational level	1.468	3.0	1.066
Marital status	1.124	1.0	1.06
Smoking status	1.532	1.0	1.238
Drinking status	1.448	1.0	1.203
Sleep duration	1.048	1.0	1.024
Physical activity	1.206	1.0	1.098
Hypertension	1.082	1.0	1.04
Dyslipdemia	1.146	1.0	1.07
BMI	1.046	1.0	1.023
LDL-C	1.083	1.0	1.04
UA	1.19	1.0	1.091

BMI - body mass index, LDL-C - low-density lipoprotein cholesterol, UA - uric acid

Table S6 Descriptive characteristics of the participants at baseline AIP-FI.

Characteristic	Total	AIP-FI				p
	(n = 4157)	Q1(n = 1039)	Q2(n = 1039)	Q3(n = 1039)	Q4(n = 1040)	
Age,years (Mean±SD)	60.0 ± 8.7	60.2 ± 8.9	59.8 ± 9.1	59.8 ± 8.4	60.3 ± 8.6	0.387
Sex,n(%)						< 0.001
Female	2486 (59.8)	551 (53)	622 (59.9)	637 (61.3)	676 (65)	
Male	1671 (40.2)	488 (47)	417 (40.1)	402 (38.7)	364 (35)	
Residence,n(%)						0.11
Urben	1331 (32.0)	306 (29.5)	358 (34.5)	331 (31.9)	336 (32.3)	
Rural	2826 (68.0)	733 (70.5)	681 (65.5)	708 (68.1)	704 (67.7)	
Educational level,n(%)						0.142
No formal education	2225 (53.6)	545 (52.5)	531 (51.2)	560 (53.9)	589 (56.7)	
Elementary school	936 (22.5)	239 (23)	235 (22.6)	237 (22.8)	225 (21.7)	
Middle and High school	707 (17.0)	193 (18.6)	194 (18.7)	161 (15.5)	159 (15.3)	
Vocational school and above	286 (6.9)	62 (6)	78 (7.5)	80 (7.7)	66 (6.4)	
Marital status,n(%)						0.227
Married	3632 (87.4)	925 (89)	910 (87.6)	900 (86.6)	897 (86.2)	
Others	525 (12.6)	114 (11)	129 (12.4)	139 (13.4)	143 (13.8)	
Smoking status,n(%)						0.004
Yes	1132 (27.2)	322 (31)	291 (28)	266 (25.6)	253 (24.3)	
No	3025 (72.8)	717 (69)	748 (72)	773 (74.4)	787 (75.7)	
Drinking status,n(%)						< 0.001
Yes	1201 (28.9)	386 (37.2)	291 (28)	268 (25.8)	256 (24.6)	
No	2956 (71.1)	653 (62.8)	748 (72)	771 (74.2)	784 (75.4)	
Sleep duration, hours (Mean ± SD)	6.2 ± 1.9	6.2 ± 1.9	6.3 ± 1.8	6.3 ± 1.9	5.9 ± 2.1	< 0.001
Physical activity (%)						< 0.001
Low	604 (34.1)	117 (27.7)	135 (29.7)	157 (37.8)	195 (40.7)	
Moderate and high	1167 (65.9)	306 (72.3)	319 (70.3)	258 (62.2)	284 (59.3)	
BMI, kg/m^2, (Median(IQR))	23.2 (20.9, 26.1)	21.8 (19.8, 23.9)	23.0 (20.6, 25.6)	23.6 (21.3, 26.3)	24.8 (22.3, 27.5)	< 0.001
FBG,mg/dL (Mean ± SD)	108.9 ± 34.0	102.4 ± 21.8	106.4 ± 30.5	108.5 ± 31.7	118.1 ± 45.7	< 0.001
TC,mg/dL (Mean ± SD)	194.0 ± 38.1	188.4 ± 34.2	192.9 ± 37.1	194.1 ± 36.4	200.5 ± 43.2	< 0.001
TG,mg/dL (Median (IQR))	106.2 (76.1, 154.9)	62.8 (53.1, 75.2)	95.6 (79.7, 119.5)	123.9 (100.9, 158.4)	180.5 (133.6, 255.1)	< 0.001
HDL-C,mg/dL (Mean ± SD)	51.2 ± 15.4	66.1 ± 15.4	52.8 ± 11.4	46.5 ± 10.2	39.6 ± 10.0	< 0.001
LDL-C,mg/dL (Mean ± SD)	116.7 ± 34.8	111.2 ± 30.6	120.6 ± 33.2	120.8 ± 34.1	114.1 ± 39.7	< 0.001
HbAlc,% (Mean ± SD)	5.3 ± 0.8	5.1 ± 0.5	5.2 ± 0.8	5.2 ± 0.8	5.4 ± 0.9	< 0.001
UA,mg/dL (Mean ± SD)	4.3 ± 1.2	4.2 ± 1.2	4.2 ± 1.2	4.4 ± 1.2	4.5 ± 1.3	< 0.001
TyG (Mean ± SD)	8.7 ± 0.7	8.1 ± 0.4	8.6 ± 0.4	8.8 ± 0.5	9.3 ± 0.7	< 0.001
eGDR (Mean ± SD)	9.3 ± 2.0	9.5 ± 1.9	9.4 ± 2.0	9.2 ± 2.0	9.0 ± 2.1	< 0.001
Hypertension,n(%)	1990 (47.9)	393 (37.8)	426 (41)	529 (50.9)	642 (61.7)	< 0.001
Dyslipedaemia,n(%)	453 (10.9)	49 (4.7)	58 (5.6)	112 (10.8)	234 (22.5)	< 0.001
Heart disease,n(%)	1398 (33.6)	286 (27.5)	291 (28)	358 (34.5)	463 (44.5)	< 0.001
Stroke,n(%)	534 (12.8)	82 (7.9)	113 (10.9)	130 (12.5)	209 (20.1)	< 0.001
DM,n(%)	1244 (29.9)	217 (20.9)	257 (24.7)	306 (29.5)	464 (44.6)	< 0.001
CMM,n(%)	612 (14.7)	86 (8.3)	105 (10.1)	135 (13)	286 (27.5)	< 0.001

BMI - body mass index, FBG - Fasting blood-glucose, TC - total cholesterol, TG - triglyceride, HDL-C - high-density lipoprotein cholesterol, LDL-C - low-density lipoprotein cholesterol, HbA1c - glycosylated hemoglobin A1c, UA - uric acid, TyG -

triglyceride-glucose, eGDR - estimated glucose disposal rate, DM - diabetes Mellitus, CMM - cardiometabolic multimorbidity, AIP - the atherogenic index of plasma, FI - frailty index, cumAIP - cumulative atherogenic index of plasma, cumFI - cumulative frailty index, IQR - interquartile range, SD - standard deviation

Table S7 Descriptive characteristics of the participants at cumulative AIP-FI.

Characteristic	Total	Cumulative AIP-FI				p
	(n = 4157)	Q1(n = 1039)	Q2(n = 1039)	Q3(n = 1039)	Q4(n = 1040)	
Age,years (Mean±SD)	60.0 ± 8.7	60.2 ± 9.0	59.5 ± 8.8	59.9 ± 8.7	60.6 ± 8.5	0.044
Sex,n(%)						< 0.001
Female	2486 (59.8)	538 (51.8)	590 (56.8)	660 (63.5)	698 (67.1)	
Male	1671 (40.2)	501 (48.2)	449 (43.2)	379 (36.5)	342 (32.9)	
Residence,n(%)						0.123
Urben	1331 (32.0)	304 (29.3)	337 (32.4)	354 (34.1)	336 (32.3)	
Rural	2826 (68.0)	735 (70.7)	702 (67.6)	685 (65.9)	704 (67.7)	
Educational level,n(%)						0.055
No formal education	2228 (53.6)	537 (51.7)	524 (50.4)	565 (54.4)	602 (57.9)	
Elementary school	936 (22.5)	243 (23.4)	242 (23.3)	233 (22.4)	218 (21)	
Middle and High school	707 (17.0)	193 (18.6)	194 (18.7)	167 (16.1)	153 (14.7)	
Vocational school and above	286 (6.9)	66 (6.4)	79 (7.6)	74 (7.1)	67 (6.4)	
Marital status,n(%)						0.061
Married	3632 (87.4)	928 (89.3)	915 (88.1)	896 (86.2)	893 (85.9)	
Others	525 (12.6)	111 (10.7)	124 (11.9)	143 (13.8)	147 (14.1)	
Smoking status,n(%)						< 0.001
Yes	1132 (27.2)	325 (31.3)	285 (27.4)	283 (27.2)	239 (23)	
No	3025 (72.8)	714 (68.7)	754 (72.6)	756 (72.8)	801 (77)	
Drinking status,n(%)						< 0.001
Yes	1201 (28.9)	390 (37.5)	306 (29.5)	274 (26.4)	231 (22.2)	
No	2956 (71.1)	649 (62.5)	733 (70.5)	765 (73.6)	809 (77.8)	
Sleep duration, hours (Mean ± SD)	6.2 ± 1.9	6.2 ± 1.9	6.3 ± 1.8	6.3 ± 2.0	5.9 ± 2.1	< 0.001
Physical activity (%)						< 0.001
Low	1480 (35.6)	285 (27.4)	349 (33.6)	391 (37.6)	455 (43.8)	
Moderate and high	2677 (64.4)	754 (72.6)	690 (66.4)	648 (62.4)	585 (56.2)	
BMI, kg/m^2, (Median(IQR))	23.2 (20.9, 26.1)	21.7 (19.8, 23.8)	23.0 (20.7, 25.6)	24.1 (21.5, 26.9)	24.7 (22.2, 27.5)	< 0.001
FBG,mg/dL (Mean ± SD)	108.9 ± 34.0	102.2 ± 21.1	106.8 ± 33.8	108.2 ± 30.0	118.3 ± 44.9	< 0.001
TC,mg/dL (Mean ± SD)	194.0 ± 38.1	187.8 ± 32.9	191.6 ± 36.7	194.3 ± 38.3	202.2 ± 42.5	< 0.001
TG,mg/dL (Median (IQR))	106.2 (76.1, 154.9)	66.4 (54.0, 82.3)	97.3 (77.9, 123.0)	124.8 (97.3, 162.8)	175.2 (127.4, 249.6)	< 0.001
HDL-C,mg/dL (Mean ± SD)	51.2 ± 15.4	65.6 ± 15.5	52.2 ± 11.2	46.9 ± 10.9	40.2 ± 11.0	< 0.001
LDL-C,mg/dL (Mean ± SD)	116.8 ± 34.9	110.1 ± 28.6	119.8 ± 33.2	120.4 ± 35.4	116.9 ± 40.5	< 0.001
HbAlc,% (Mean ± SD)	5.3 ± 0.8	5.1 ± 0.5	5.2 ± 0.8	5.2 ± 0.8	5.4 ± 0.9	< 0.001
UA,mg/dL (Mean ± SD)	4.3 ± 1.2	4.2 ± 1.2	4.2 ± 1.2	4.4 ± 1.2	4.5 ± 1.3	< 0.001
TyG (Mean ± SD)	8.7 ± 0.7	8.1 ± 0.4	8.5 ± 0.4	8.8 ± 0.5	9.2 ± 0.7	< 0.001
eGDR (Mean ± SD)	9.3 ± 2.0	9.5 ± 1.9	9.3 ± 2.0	9.2 ± 2.0	9.0 ± 2.1	< 0.001
Hypertension,n(%)	1990 (47.9)	366 (35.2)	437 (42.1)	531 (51.1)	656 (63.1)	< 0.001
Dyslipedaemia,n(%)	453 (10.9)	35 (3.4)	66 (6.4)	118 (11.4)	234 (22.5)	< 0.001
Heart disease,n(%)	1398 (33.6)	258 (24.8)	262 (25.2)	388 (37.3)	490 (47.1)	< 0.001
Stroke,n(%)	534 (12.8)	66 (6.4)	93 (9)	147 (14.1)	228 (21.9)	< 0.001
DM,n(%)	1244 (29.9)	188 (18.1)	235 (22.6)	331 (31.9)	490 (47.1)	< 0.001
CMM,n(%)	612 (14.7)	56 (5.4)	84 (8.1)	169 (16.3)	303 (29.1)	< 0.001

BMI - body mass index, FBG - Fasting blood-glucose, TC - total cholesterol, TG - triglyceride, HDL-C - high-density lipoprotein cholesterol, LDL-C - low-density lipoprotein cholesterol, HbA1c - glycosylated hemoglobin A1c, UA - uric acid, TyG -

triglyceride-glucose, eGDR - estimated glucose disposal rate, DM - diabetes Mellitus, CMM - cardiometabolic multimorbidity, AIP - the atherogenic index of plasma, FI - frailty index, cumAIP - cumulative atherogenic index of plasma, cumFI - cumulative frailty index, IQR - interquartile range, SD - standard deviation

Table S8 Descriptive characteristics of the participants at clustering of AIP-FI.

Characteristic	Total (n = 4157)	Clustering of AIP-FI Q1(n = 3386)	Q2(n = 771)	p
Age,years (Mean±SD)	60.0 ± 8.7	59.9 ± 8.8	60.7 ± 8.5	0.030
Sex,n(%)				< 0.001
Female	2486 (59.8)	1968 (58.1)	518 (67.2)	
Male	1671 (40.2)	1418 (41.9)	253 (32.8)	
Residence,n(%)				0.922
Urben	1331 (32.0)	1083 (32)	248 (32.2)	
Rural	2826 (68.0)	2303 (68)	523 (67.8)	
Educational level,n(%)				0.015
No formal education	2228 (53.6)	1777 (52.5)	451 (58.5)	
Elementary school	936 (22.5)	771 (22.8)	165 (21.4)	
Middle and High school	707 (17.0)	598 (17.7)	109 (14.1)	
Vocational school and above	286 (6.9)	240 (7.1)	46 (6)	
Marital status,n(%)				0.3
Married	3632 (87.4)	2967 (87.6)	665 (86.3)	
Others	525 (12.6)	419 (12.4)	106 (13.7)	
Smoking status,n(%)				0.002
Yes	1132 (27.2)	956 (28.2)	176 (22.8)	
No	3025 (72.8)	2430 (71.8)	595 (77.2)	
Drinking status,n(%)				< 0.001
Yes	1201 (28.9)	1039 (30.7)	162 (21)	
No	2956 (71.1)	2347 (69.3)	609 (79)	
Sleep duration, hours (Mean ± SD)	6.2 ± 1.9	6.3 ± 1.9	5.8 ± 2.0	< 0.001
Physical activity (%)				< 0.001
Low	1480 (35.6)	1126 (33.3)	354 (45.9)	
Moderate and high	2677 (64.4)	2260 (66.7)	417 (54.1)	
BMI, kg/m^2, (Median(IQR))	23.2 (20.9, 26.1)	23.0 (20.7, 25.7)	24.9 (22.2, 27.8)	< 0.001
FBG,mg/dL (Mean ± SD)	108.9 ± 34.0	106.2 ± 29.2	120.5 ± 48.2	< 0.001
TC,mg/dL (Mean ± SD)	194.0 ± 38.1	191.9 ± 36.1	203.2 ± 44.7	< 0.001
TG,mg/dL (Median (IQR))	106.2 (76.1, 154.9)	96.5 (71.7, 131.9)	185.9 (134.5, 266.4)	< 0.001
HDL-C,mg/dL (Mean ± SD)	51.2 ± 15.4	54.0 ± 15.0	39.3 ± 10.8	< 0.001
LDL-C,mg/dL (Mean ± SD)	116.8 ± 34.9	117.0 ± 33.3	115.8 ± 41.5	0.357
HbAlc,% (Mean ± SD)	5.3 ± 0.8	5.2 ± 0.7	5.4 ± 1.0	< 0.001
UA,mg/dL (Mean ± SD)	4.3 ± 1.2	4.3 ± 1.2	4.6 ± 1.3	< 0.001
TyG (Mean ± SD)	8.7 ± 0.7	8.5 ± 0.5	9.3 ± 0.7	< 0.001
eGDR (Mean ± SD)	9.3 ± 2.0	9.3 ± 2.0	9.0 ± 2.1	< 0.001
Hypertension,n(%)	1990 (47.9)	1480 (43.7)	510 (66.1)	< 0.001
Dyslipedaemia,n(%)	453 (10.9)	249 (7.4)	204 (26.5)	< 0.001
Heart disease,n(%)	1398 (33.6)	1030 (30.4)	368 (47.7)	< 0.001
Stroke,n(%)	534 (12.8)	357 (10.5)	177 (23)	< 0.001
DM,n(%)	1244 (29.9)	863 (25.5)	381 (49.4)	< 0.001
CMM,n(%)	612 (14.7)	375 (11.1)	237 (30.7)	< 0.001

BMI - body mass index, FBG - Fasting blood-glucose, TC - total cholesterol, TG - triglyceride, HDL-C - high-density lipoprotein cholesterol, LDL-C - low-density lipoprotein cholesterol, HbA1c - glycosylated hemoglobin A1c, UA - uric acid, TyG - triglyceride-glucose, eGDR - estimated glucose disposal rate, DM - diabetes Mellitus, CMM - cardiometabolic multimorbidity, AIP - the atherogenic index of plasma, FI - frailty index, cumAIP - cumulative atherogenic index of plasma, cumFI - cumulative frailty index,

IQR - interquartile range, SD - standard deviation

Table S9 Association of baseline and longitudinal AIP-FI trajectories with the incidence of heart disease, stroke and DM

Variable	n.event_%	Model 1		Model 2		Model 3	
		HR (95%CI)	P value	HR (95%CI)	P value	HR (95%CI)	P value
**Heart disease**							
AIP-FI (per 1 SD)	1398 (33.6)	1.18 (1.13~1.23)	<0.001	1.18 (1.13~1.24)	<0.001	1.12 (1.07~1.18)	<0.001
AIP-FI quartile							
Q1	286 (27.5)	1(Ref)		1(Ref)		1(Ref)	
Q2	291 (28)	0.99 (0.84~1.16)	0.876	0.96 (0.82~1.14)	0.664	0.94 (0.8~1.11)	0.488
Q3	358 (34.5)	1.31 (1.13~1.54)	0.001	1.29 (1.11~1.51)	0.001	1.19 (1.01~1.39)	0.034
Q4	463 (44.5)	1.69 (1.45~1.96)	<0.001	1.67 (1.44~1.93)	<0.001	1.42 (1.21~1.65)	<0.001
cumAIP-FI(per 1 SD)	1398 (33.6)	1.24 (1.19~1.3)	<0.001	1.25 (1.2~1.3)	<0.001	1.19 (1.13~1.25)	<0.001
cumAIP-FI quartile							
Q1	254 (24.4)	1(Ref)		1(Ref)		1(Ref)	
Q2	276 (26.6)	1.1 (0.93~1.3)	0.278	1.09 (0.92~1.29)	0.324	1.04 (0.88~1.24)	0.623
Q3	376 (36.2)	1.56 (1.33~1.83)	<0.001	1.56 (1.32~1.83)	<0.001	1.42 (1.2~1.67)	<0.001
Q4	492 (47.3)	2.05 (1.76~2.39)	<0.001	2.05 (1.75~2.39)	<0.001	1.73 (1.47~2.04)	<0.001
K-means Group							
Cluster1	1030 (30.4)	1(Ref)		1(Ref)		1(Ref)	
Cluster2	368 (47.7)	1.61 (1.43~1.82)	<0.001	1.55 (1.38~1.75)	<0.001	1.36 (1.2~1.55)	<0.001
**Stroke**							
AIP-FI (per 1 SD)	534 (12.8)	1.34 (1.26~1.42)	<0.001	1.34 (1.26~1.43)	<0.001	1.22 (1.14~1.31)	<0.001
AIP-FI quartile							
Q1	82 (7.9)	1(Ref)		1(Ref)		1(Ref)	
Q2	113 (10.9)	1.38 (1.04~1.83)	0.027	1.38 (1.04~1.84)	0.025	1.33 (1~1.77)	0.053
Q3	130 (12.5)	1.69 (1.28~2.23)	<0.001	1.69 (1.28~2.24)	<0.001	1.45 (1.1~1.93)	0.009
Q4	209 (20.1)	2.66 (2.06~3.44)	<0.001	2.67 (2.06~3.45)	<0.001	1.95 (1.49~2.54)	<0.001
cumAIP-FI(per 1 SD)	534 (12.8)	1.45 (1.37~1.53)	<0.001	1.46 (1.38~1.54)	<0.001	1.35 (1.27~1.44)	<0.001
cumAIP-FI quartile							
Q1	70 (6.7)	1(Ref)		1(Ref)		1(Ref)	
Q2	91 (8.8)	1.35 (0.99~1.84)	0.062	1.34 (0.98~1.84)	0.063	1.23 (0.9~1.68)	0.197
Q3	139 (13.4)	2.15 (1.61~2.86)	<0.001	2.17 (1.63~2.9)	<0.001	1.83 (1.36~2.45)	<0.001
Q4	234 (22.5)	3.6 (2.75~4.71)	<0.001	3.62 (2.76~4.74)	<0.001	2.66 (2.01~3.54)	<0.001
K-means Group							
Cluster1	357 (10.5)	1(Ref)		1(Ref)		1(Ref)	
Cluster2	177 (23)	2.24 (1.87~2.68)	<0.001	2.07 (1.72~2.49)	<0.001	1.67 (1.37~2.03)	<0.001
**DM**							
AIP-FI (per 1 SD)	1244 (29.9)	1.29 (1.23~1.34)	<0.001	1.29 (1.23~1.34)	<0.001	1.2 (1.14~1.26)	<0.001
AIP-FI quartile							
Q1	217 (20.9)	1(Ref)		1(Ref)		1(Ref)	
Q2	257 (24.7)	1.17 (0.98~1.4)	0.086	1.17 (0.97~1.4)	0.095	1.12 (0.93~1.34)	0.232
Q3	306 (29.5)	1.5 (1.26~1.79)	<0.001	1.49 (1.25~1.78)	<0.001	1.31 (1.1~1.56)	0.003
Q4	464 (44.6)	2.28 (1.94~2.68)	<0.001	2.27 (1.93~2.67)	<0.001	1.8 (1.52~2.13)	<0.001
cumAIP-FI(per 1 SD)	1244 (29.9)	1.37 (1.32~1.42)	<0.001	1.37 (1.32~1.43)	<0.001	1.29 (1.23~1.35)	<0.001
cumAIP-FI quartile							
Q1	193 (18.6)	1(Ref)		1(Ref)		1(Ref)	
Q2	233 (22.4)	1.24 (1.02~1.5)	0.029	1.23 (1.02~1.49)	0.033	1.15 (0.95~1.4)	0.151
Q3	321 (30.9)	1.78 (1.49~2.13)	<0.001	1.78 (1.49~2.13)	<0.001	1.55 (1.29~1.86)	<0.001
Q4	497 (47.8)	2.81 (2.38~3.32)	<0.001	2.81 (2.37~3.32)	<0.001	2.24 (1.88~2.67)	<0.001
K-means Group							

Cluster1	863 (25.5)	1(Ref)		1(Ref)		1(Ref)	
Cluster2	381 (49.4)	2.04 (1.81~2.31)	<0.001	1.98 (1.75~2.24)	<0.001	1.65 (1.44~1.87)	<0.001

Model 1 adjusted for age, sex; Model 2 adjusted for variables in Model 1 and residence, educational level, marital status, smoking status, and drinking status; Model 3 adjusted for variables in Model 2 and sleep duration, physical activity, BMI, history of hypertension and dyslipdemia, LDL-C and UA.

HR - hazard ratio, CI - confidence interval, Ref - reference

Table S10 Predictive value of AIP-FI for incident CMM.

Variable	AUC(95%CI)
CumAIP-FI	71.17% (68.93% ~ 73.42%)
AIP-FI	66.26% (63.83% ~ 68.68%)
CumAIP	61.10% (58.76% ~ 63.45%)
TyG	61.33% (58.93% ~ 63.74%)
eGDR	57.12% (54.73% ~ 59.50%)

cumAIP-FI - cumulative atherogenic index of plasma-frailty index, AIP-FI - atherogenic index of plasma-frailty index, cumAIP - cumulative atherogenic index of plasma, TyG - triglyceride glucose, eGDR - estimated glucose disposal rate

Table S11 Incremental predictive value of baseline and cumAIP-FI for CMM.

Model	NRI (95% CI)	P value	IDI (95% CI)	P value
Basic model	Ref	Ref	Ref	Ref
+cumAIP-FI	0.157 (0.04-0.243)	0.004	0.024 (0.007-0.046)	<0.001
+AIP-FI	0.025 (-0.057-0.124)	0.435	0.011 (-0.002-0.03)	0.116
+cumAIP	0.118 (0.008-0.217)	0.024	0.004 (-0.003-0.014)	0.303
+TyG	0.093 (0.001-0.188)	0.044	0.007 (-0.004-0.024)	0.204
+eGDR	-0.009 (-0.078-0.14)	1.21	0.011 (0.001-0.025)	0.016

The basic model included age, sex, residence, educational level, marital status, smoking status, and drinking status, hypertension, dyslipidemia, hs-CRP, LDL-C and UA.

NRI - net reclassification improvement, Ref - reference, IDI - integrated discrimination improvement, CI - confidence interval, Ref - reference, cumAIP-FI - cumulative atherogenic index of plasma-frailty index, AIP-FI - atherogenic index of plasma-frailty index, cumAIP - cumulative atherogenic index of plasma, TyG - triglyceride glucose, eGDR - estimated glucose disposal rate

Table S12 Cox regression for the association of baseline and cumulative AIP-FI with the incidence of CMM by age group.

Variable	n.event_%	Model 1		Model 2		Model 3	
		HR (95%CI)	P value	HR (95%CI)	P value	HR (95%CI)	P value
**Age < 60 years**							
**CMM**							
AIP-FI (per 1 SD)	262 (12.7)	1.48 (1.39~1.59)	<0.001	1.49 (1.38~1.61)	<0.001	1.42 (1.31~1.55)	<0.001
AIP-FI quartile							
Q1	28 (5.4)	1(Ref)		1(Ref)		1(Ref)	
Q2	41 (7.9)	1.49 (0.92~2.4)	0.107	1.39 (0.86~2.26)	0.177	1.32 (0.81~2.14)	0.26
Q3	58 (11.2)	2.29 (1.46~3.6)	<0.001	2.12 (1.35~3.34)	0.001	1.7 (1.08~2.7)	0.023
Q4	135 (26.1)	5.22 (3.47~7.85)	<0.001	4.67 (3.1~7.05)	<0.001	3.39 (2.22~5.18)	<0.001
cumAIP-FI(per 1 SD)	262 (12.7)	1.54 (1.46~1.63)	<0.001	1.6 (1.5~1.7)	<0.001	1.52 (1.41~1.64)	<0.001
cumAIP-FI quartile							
Q1	20 (3.9)	1(Ref)		1(Ref)		1(Ref)	
Q2	36 (7)	1.83 (1.06~3.17)	0.03	1.78 (1.03~3.08)	0.039	1.69 (0.97~2.92)	0.062
Q3	57 (11)	3.01 (1.81~5.01)	<0.001	2.8 (1.68~4.68)	<0.001	2.32 (1.38~3.89)	0.001
Q4	149 (28.8)	8.24 (5.16~13.15)	<0.001	7.59 (4.74~12.15)	<0.001	5.53 (3.41~8.97)	<0.001
**Heart disease**							
AIP-FI (per 1 SD)	608 (29.4)	1.21 (1.14~1.28)	<0.001	1.2 (1.13~1.28)	<0.001	1.16 (1.08~1.24)	<0.001
AIP-FI quartile							
Q1	112 (21.7)	1(Ref)		1(Ref)		1(Ref)	
Q2	127 (24.6)	1.15 (0.89~1.48)	0.29	1.12 (0.86~1.44)	0.4	1.08 (0.83~1.4)	0.565
Q3	159 (30.8)	1.57 (1.23~2)	<0.001	1.54 (1.2~1.96)	0.001	1.39 (1.08~1.78)	0.01
Q4	210 (40.6)	2.03 (1.61~2.56)	<0.001	1.94 (1.54~2.45)	<0.001	1.67 (1.31~2.13)	<0.001
cumAIP-FI(per	608 (29.4)	1.27 (1.2~1.35)	<0.001	1.28	<0.001	1.23	<0.001

1 SD)				(1.21~1.36)		(1.15~1.32<br>)	
cumAIP-FI quartile							
Q1	98 (19)	1(Ref)		1(Ref)		1(Ref)	
Q2	121 (23.4)	1.27 (0.97~1.66)	0.077	1.27 (0.97~1.66)	0.08	1.23 (0.94~1.61<br>)	0.129
Q3	154 (29.8)	1.66 (1.29~2.14)	<0.001	1.63 (1.26~2.1)	<0.001	1.49 (1.15~1.94<br>)	0.002
Q4	235 (45.5)	2.65 (2.09~3.36)	<0.001	2.57 (2.02~3.26)	<0.001	2.23 (1.74~2.87<br>)	<0.001
**Stroke**							
AIP-FI (per 1 SD)	216 (10.4)	1.4 (1.29~1.52)	<0.001	1.38 (1.26~1.51)	<0.001	1.3 (1.17~1.44<br>)	<0.001
AIP-FI quartile							
Q1	26 (5)	1(Ref)		1(Ref)		1(Ref)	
Q2	45 (8.7)	1.78 (1.1~2.88)	0.02	1.66 (1.02~2.7)	0.041	1.62 (1~2.63)	0.052
Q3	58 (11.2)	2.48 (1.56~3.94)	<0.001	2.24 (1.4~3.57)	0.001	1.95 (1.22~3.12<br>)	0.005
Q4	87 (16.8)	3.57 (2.31~5.54)	<0.001	3.05 (1.95~4.75)	<0.001	2.3 (1.45~3.64<br>)	<0.001
cumAIP-FI(per 1 SD)	216 (10.4)	1.45 (1.35~1.56)	<0.001	1.46 (1.34~1.59)	<0.001	1.37 (1.25~1.51<br>)	<0.001
cumAIP-FI quartile							
Q1	22 (4.3)	1(Ref)		1(Ref)		1(Ref)	
Q2	36 (7)	1.66 (0.98~2.83)	0.06	1.57 (0.92~2.67)	0.097	1.54 (0.9~2.63)	0.111
Q3	58 (11.2)	2.81 (1.72~4.59)	<0.001	2.5 (1.53~4.1)	<0.001	2.2 (1.33~3.61<br>)	0.002
Q4	100 (19.3)	4.96 (3.13~7.88)	<0.001	4.35 (2.73~6.93)	<0.001	3.41 (2.1~5.52)	<0.001
**DM**							
AIP-FI (per 1 SD)	584 (28.3)	1.32 (1.25~1.39)	<0.001	1.3 (1.23~1.38)	<0.001	1.24 (1.17~1.33<br>)	<0.001
AIP-FI quartile							
Q1	101 (19.5)	1(Ref)		1(Ref)		1(Ref)	
Q2	112 (21.7)	1.12 (0.86~1.47)	0.41	1.11 (0.85~1.46)	0.445	1.07 (0.82~1.41	0.607

						)	
Q3	144 (27.9)	1.58 (1.23~2.04)	<0.001	1.55 (1.2~2.01)	0.001	1.37 (1.05~1.77)	0.019
Q4	227 (43.9)	2.48 (1.96~3.14)	<0.001	2.41 (1.9~3.06)	<0.001	1.97 (1.54~2.52)	<0.001
cumAIP-FI(per 1 SD)	584 (28.3)	1.37 (1.3~1.44)	<0.001	1.37 (1.29~1.44)	<0.001	1.3 (1.23~1.38)	<0.001
cumAIP-FI quartile							
Q1	81 (15.7)	1(Ref)		1(Ref)		1(Ref)	
Q2	112 (21.7)	1.43 (1.07~1.9)	0.014	1.42 (1.07~1.89)	0.017	1.38 (1.04~1.84)	0.027
Q3	151 (29.2)	2.02 (1.55~2.65)	<0.001	1.99 (1.52~2.62)	<0.001	1.79 (1.36~2.35)	<0.001
Q4	240 (46.4)	3.36 (2.61~4.33)	<0.001	3.29 (2.55~4.25)	<0.001	2.73 (2.09~3.56)	<0.001
**Age ≥ 60 years**							
**CMM**							
AIP-FI (per 1 SD)	350 (16.7)	1.36 (1.25~1.47)	<0.001	1.31 (1.21~1.43)	<0.001	1.21 (1.11~1.33)	<0.001
AIP-FI quartile							
Q1	60 (11.5)	1(Ref)		1(Ref)		1(Ref)	
Q2	63 (12.1)	1.01 (0.71~1.44)	0.966	1.01 (0.71~1.44)	0.971	0.93 (0.65~1.33)	0.691
Q3	78 (14.9)	1.3 (0.93~1.82)	0.13	1.25 (0.89~1.75)	0.201	1.05 (0.74~1.47)	0.8
Q4	149 (28.5)	2.41 (1.78~3.26)	<0.001	2.27 (1.67~3.09)	<0.001	1.7 (1.24~2.34)	0.001
cumAIP-FI(per 1 SD)	350 (16.7)	1.54 (1.42~1.66)	<0.001	1.52 (1.4~1.65)	<0.001	1.41 (1.29~1.54)	<0.001
cumAIP-FI quartile							
Q1	37 (7.1)	1(Ref)		1(Ref)		1(Ref)	
Q2	58 (11.1)	1.61 (1.06~2.43)	0.025	1.57 (1.04~2.38)	0.032	1.39 (0.92~2.12)	0.12
Q3	93 (17.8)	2.58 (1.76~3.79)	<0.001	2.53 (1.72~3.72)	<0.001	2.06 (1.39~3.04)	<0.001
Q4	162 (31)	4.43 (3.09~6.37)	<0.001	4.23 (2.93~6.11)	<0.001	3.19 (2.19~4.66)	<0.001
**Heart disease**							
AIP-FI (per 1 SD)	790 (37.8)	1.16 (1.09~1.24)	<0.001	1.15 (1.08~1.22)	<0.001	1.1 (1.03~1.18)	0.007
AIP-FI quartile							

Q1	175 (33.5)	1(Ref)		1(Ref)		1(Ref)	
Q2	162 (31)	0.89 (0.72~1.1)	0.269	0.87 (0.7~1.08)	0.203	0.85 (0.68~1.05)	0.132
Q3	204 (39.1)	1.2 (0.98~1.47)	0.084	1.15 (0.93~1.41)	0.193	1.06 (0.87~1.31)	0.555
Q4	249 (47.6)	1.44 (1.18~1.75)	<0.001	1.38 (1.13~1.69)	0.001	1.21 (0.99~1.49)	0.066
cumAIP-FI(per 1 SD)	790 (37.8)	1.22 (1.14~1.29)	<0.001	1.2 (1.13~1.28)	<0.001	1.15 (1.07~1.23)	<0.001
cumAIP-FI quartile							
Q1	153 (29.3)	1(Ref)		1(Ref)		1(Ref)	
Q2	160 (30.7)	1.06 (0.85~1.33)	0.597	1.03 (0.83~1.29)	0.766	0.99 (0.79~1.25)	0.961
Q3	221 (42.3)	1.53 (1.24~1.89)	<0.001	1.51 (1.22~1.86)	<0.001	1.38 (1.11~1.71)	0.003
Q4	256 (48.9)	1.74 (1.42~2.14)	<0.001	1.68 (1.37~2.07)	<0.001	1.48 (1.19~1.84)	<0.001
**Stoke**							
AIP-FI (per 1 SD)	318 (15.2)	1.28 (1.17~1.4)	<0.001	1.26 (1.15~1.38)	<0.001	1.18 (1.07~1.3)	0.001
AIP-FI quartile							
Q1	54 (10.3)	1(Ref)		1(Ref)		1(Ref)	
Q2	70 (13.4)	1.28 (0.89~1.82)	0.179	1.3 (0.91~1.86)	0.145	1.22 (0.85~1.75)	0.278
Q3	74 (14.2)	1.41 (0.99~2.01)	0.055	1.41 (0.99~2.01)	0.056	1.24 (0.87~1.78)	0.237
Q4	120 (22.9)	2.25 (1.63~3.12)	<0.001	2.2 (1.58~3.05)	<0.001	1.75 (1.24~2.47)	0.001
cumAIP-FI(per 1 SD)	318 (15.2)	1.45 (1.33~1.58)	<0.001	1.44 (1.32~1.57)	<0.001	1.36 (1.23~1.5)	<0.001
cumAIP-FI quartile							
Q1	46 (8.8)	1(Ref)		1(Ref)		1(Ref)	
Q2	59 (11.3)	1.32 (0.9~1.95)	0.157	1.34 (0.91~1.97)	0.144	1.21 (0.82~1.79)	0.347
Q3	81 (15.5)	1.87 (1.3~2.69)	0.001	1.89 (1.31~2.73)	0.001	1.65 (1.13~2.4)	0.009
Q4	132 (25.2)	3.01 (2.14~4.23)	<0.001	2.94 (2.08~4.17)	<0.001	2.39 (1.66~3.44)	<0.001
**DM**							
AIP-FI (per 1 SD)	660 (31.6)	1.25 (1.18~1.34)	<0.001	1.24 (1.16~1.32)	<0.001	1.15 (1.08~1.24)	<0.001
AIP-FI quartile	AIP_FI_2011.z.4	AIP_FI_2011.z.4	AIP_FI_2011.z.4	AIP_FI_2011.z.4	AIP_FI_2011.z.4	AIP_FI_2011.z.4	AIP_FI_2011.z.4
Q1	120 (22.9)	1(Ref)		1(Ref)		1(Ref)	
Q2	143 (27.4)	1.17 (0.92~1.49)	0.21	1.16 (0.91~1.48)	0.223	1.09 (0.86~1.4)	0.473
Q3	159 (30.5)	1.37	0.009	1.33	0.019	1.18	0.177

		(1.08~1.74)		(1.05~1.69)		(0.93~1.5)	
Q4	238 (45.5)	2.03 (1.63~2.54)	<0.001	1.96 (1.57~2.46)	<0.001	1.58 (1.25~2)	<0.001
cumAIP-FI(per 1 SD)	660 (31.6)	1.37 (1.29~1.46)	<0.001	1.36 (1.28~1.45)	<0.001	1.27 (1.18~1.36)	<0.001
cumAIP-FI quartile							
Q1	110 (21)	1(Ref)		1(Ref)		1(Ref)	
Q2	124 (23.8)	1.16 (0.89~1.49)	0.272	1.13 (0.87~1.46)	0.366	1.03 (0.79~1.34)	0.819
Q3	169 (32.4)	1.62 (1.27~2.07)	<0.001	1.59 (1.25~2.03)	<0.001	1.39 (1.08~1.78)	0.01
Q4	257 (49.1)	2.47 (1.97~3.1)	<0.001	2.39 (1.9~3.01)	<0.001	1.94 (1.53~2.47)	<0.001

Model 1 adjusted for age, sex; Model 2 adjusted for variables in Model 1 and residence, educational level, marital status, smoking status, and drinking status; Model 3 adjusted for variables in Model 2 and sleep duration, physical activity, BMI, history of hypertension and dyslipdemia, LDL-C and UA.

HR - hazard ratio, CI - confidence interval, Ref - reference, LDL-C - low-density lipoprotein cholesterol, UA - uric acid

Table S13 Logical analysis for the association between baseline and cumulative AIP-FI with the incidence of CMM.

Variable	n.event_%	Model 1		Model 2		Model 3	
		OR (95%CI)	P value	OR (95%CI)	P value	OR (95%CI)	P value
**CMM**							
AIP-FI (per 1 SD)	612 (14.7)	1.63 (1.51~1.77)	<0.001	1.64 (1.52~1.78)	<0.001	1.48 (1.36~1.61)	<0.001
AIP-FI quartile							
Q1	86 (8.3)	1(Ref)		1(Ref)		1(Ref)	
Q2	105 (10.1)	1.24 (0.92~1.67)	0.164	1.22 (0.9~1.65)	0.195	1.14 (0.84~1.54)	0.406
Q3	135 (13)	1.64 (1.23~2.18)	0.001	1.62 (1.22~2.16)	0.001	1.31 (0.98~1.76)	0.072
Q4	286 (27.5)	4.12 (3.18~5.35)	<0.001	4.11 (3.17~5.34)	<0.001	2.93 (2.22~3.85)	<0.001
cumAIP-FI(per 1 SD)	612 (14.7)	1.9 (1.75~2.06)	<0.001	1.91 (1.76~2.07)	<0.001	1.71 (1.57~1.86)	<0.001
cumAIP-FI quartile							
Q1	56 (5.4)	1(Ref)		1(Ref)		1(Ref)	
Q2	84 (8.1)	1.55 (1.09~2.2)	0.014	1.53 (1.08~2.17)	0.017	1.36 (0.96~1.94)	0.088
Q3	169 (16.3)	3.39 (2.47~4.65)	<0.001	3.41 (2.48~4.68)	<0.001	2.77 (2.01~3.83)	<0.001
Q4	303 (29.1)	7.12 (5.26~9.63)	<0.001	7.16 (5.29~9.7)	<0.001	5.04 (3.68~6.9)	<0.001
**Heart disease**							
AIP-FI (per 1 SD)	1398 (33.6)	1.31 (1.22~1.39)	<0.001	1.31 (1.23~1.4)	<0.001	1.23 (1.15~1.32)	<0.001
AIP-FI quartile							
Q1	286 (27.5)	1(Ref)		1(Ref)		1(Ref)	
Q2	291 (28)	1.02 (0.84~1.23)	0.877	0.99 (0.82~1.2)	0.935	0.96 (0.79~1.17)	0.713
Q3	358 (34.5)	1.37 (1.14~1.65)	0.001	1.35 (1.12~1.63)	0.002	1.25 (1.03~1.51)	0.026

Q4	463 (44.5)	2.06 (1.72~2.48)	<0.001	2.05 (1.71~2.47)	<0.001	1.73 (1.42~2.1)	<0.001
cumAIP-FI(per 1 SD)	1398 (33.6)	1.44 (1.34~1.54)	<0.001	1.44 (1.35~1.54)	<0.001	1.35 (1.26~1.45)	<0.001
cumAIP-FI quartile							
Q1	258 (24.8)	1(Ref)		1(Ref)		1(Ref)	
Q2	262 (24.8)	1.02 (0.84~1.25)	0.821	1.01 (0.82~1.23)	0.953	0.95 (0.77~1.16)	0.601
Q3	388 (37.3)	1.78 (1.47~2.15)	<0.001	1.77 (1.46~2.14)	<0.001	1.62 (1.34~1.98)	<0.001
Q4	490 (47.1)	2.62 (2.17~3.17)	<0.001	2.62 (2.17~3.17)	<0.001	2.22 (1.82~2.71)	<0.001
**Stroke**							
AIP-FI (per 1 SD)	534 (12.8)	1.44 (1.33~1.56)	<0.001	1.45 (1.34~1.56)	<0.001	1.31 (1.21~1.43)	<0.001
AIP-FI quartile							
Q1	82 (7.9)	1(Ref)		1(Ref)		1(Ref)	
Q2	113 (10.9)	1.45 (1.07~1.95)	0.016	1.46 (1.08~1.97)	0.014	1.4 (1.04~1.9)	0.029
Q3	130 (12.5)	1.7 (1.27~2.28)	<0.001	1.71 (1.27~2.29)	<0.001	1.46 (1.08~1.97)	0.013
Q4	209 (20.1)	2.98 (2.27~3.92)	<0.001	2.99 (2.27~3.93)	<0.001	2.22 (1.67~2.96)	<0.001
cumAIP-FI(per 1 SD)	534 (12.8)	1.6 (1.47~1.73)	<0.001	1.6 (1.48~1.74)	<0.001	1.45 (1.33~1.58)	<0.001
cumAIP-FI quartile							
Q1	66 (6.4)	1(Ref)		1(Ref)		1(Ref)	
Q2	93 (9)	1.49 (1.07~2.07)	0.018	1.49 (1.07~2.07)	0.018	1.39 (0.99~1.93)	0.055
Q3	147 (14.1)	2.5 (1.84~3.4)	<0.001	2.55 (1.87~3.46)	<0.001	2.19 (1.6~3)	<0.001
Q4	228 (21.9)	4.26 (3.18~5.7)	<0.001	4.29 (3.2~5.76)	<0.001	3.22 (2.37~4.37)	<0.001
**DM**							
AIP-FI (per 1 SD)	1244 (29.9)	1.52 (1.42~1.62)	<0.001	1.51 (1.41~1.62)	<0.001	1.39 (1.29~1.49)	<0.001
AIP-FI quartile							
Q1	217 (20.9)	1(Ref)		1(Ref)		1(Ref)	
Q2	257 (24.7)	1.24 (1.01~1.52)	0.041	1.24 (1.01~1.52)	0.044	1.18 (0.96~1.46)	0.12
Q3	306 (29.5)	1.57 (1.29~1.92)	<0.001	1.56 (1.28~1.91)	<0.001	1.36 (1.1~1.67)	0.004
Q4	464 (44.6)	3.01 (2.48~3.66)	<0.001	3 (2.47~3.65)	<0.001	2.36 (1.93~2.89)	<0.001
cumAIP-FI(per 1 SD)	1244 (29.9)	1.72 (1.6~1.85)	<0.001	1.72 (1.6~1.85)	<0.001	1.58 (1.46~1.7)	<0.001
cumAIP-FI quartile							
Q1	188 (18.1)	1(Ref)		1(Ref)		1(Ref)	
Q2	235 (22.6)	1.33 (1.07~1.64)	0.01	1.32 (1.07~1.64)	0.011	1.23 (0.99~1.53)	0.065
Q3	331 (31.9)	2.11 (1.72~2.59)	<0.001	2.11 (1.72~2.6)	<0.001	1.83 (1.48~2.26)	<0.001
Q4	490 (29.9)	4 (3.27~4.89)	<0.001	4 (3.27~4.89)	<0.001	3.13 (2.54~3.87)	<0.001

Model 1 adjusted for age, sex; Model 2 adjusted for variables in Model 1 and residence, educational level, marital status, smoking status, and drinking status; Model 3 adjusted for variables in Model 2 and sleep duration, physical activity, BMI, history of hypertension and dyslipdemia, LDL-C and UA.

HR - hazard ratio, CI - confidence interval, Ref - reference, LDL-C - low-density lipoprotein cholesterol, UA - uric acid

Table S14 Cox regression analysis of the association between baseline and cumulative AIP-FI with the incidence of CMM after excluding participants with missing values (n=846).

Variable	n.event_%	Model 1		Model 2		Model 3	
		HR (95%CI)	P value	HR (95%CI)	P value	HR (95%CI)	P value
**CMM**							
AIP-FI (per 1 SD)	139 (16.4)	1.73 (1.52~1.98)	<0.001	1.74 (1.52~1.99)	<0.001	1.53 (1.31~1.78)	<0.001
AIP-FI quartile							
Q1	17 (8.3)	1(Ref)		1(Ref)		1(Ref)	
Q2	18 (8)	0.82 (0.39~1.69)	0.583	0.84 (0.4~1.75)	0.637	0.77 (0.36~1.65)	0.503
Q3	36 (18.5)	2.4 (1.29~4.46)	0.006	2.29 (1.23~4.29)	0.009	2.1 (1.12~3.95)	0.021
Q4	68 (30.8)	5.02 (2.85~8.85)	<0.001	5.05 (2.85~8.94)	<0.001	3.49 (1.92~6.34)	<0.001
cumAIP-FI(per 1 SD)	139 (16.4)	1.68 (1.49~1.89)	<0.001	1.74 (1.53~1.98)	<0.001	1.59 (1.37~1.85)	<0.001
cumAIP-FI quartile							
Q1	7 (3.3)	1(Ref)		1(Ref)		1(Ref)	
Q2	17 (8.3)	2.58 (0.99~6.72)	0.052	2.57 (0.98~6.7)	0.054	2.09 (0.79~5.55)	0.138
Q3	44 (21.7)	7.51 (3.18~17.75)	<0.001	7.25 (3.06~17.17)	<0.001	5.81 (2.44~13.83)	<0.001
Q4	70 (31.5)	12.25 (5.29~28.39)	<0.001	12.02 (5.17~27.91)	<0.001	7.67 (3.24~18.18)	<0.001
**Heart disease**							
AIP-FI (per 1 SD)	310 (36.6)	1.35 (1.21~1.5)	<0.001	1.36 (1.22~1.52)	<0.001	1.29 (1.14~1.46)	<0.001
AIP-FI quartile							
Q1	64 (31.1)	1(Ref)		1(Ref)		1(Ref)	
Q2	59 (26.3)	0.77 (0.52~1.13)	0.184	0.75 (0.51~1.1)	0.139	0.72 (0.48~1.07)	0.101
Q3	83 (42.6)	1.48 (1.04~2.11)	0.03	1.37 (0.95~1.96)	0.088	1.29 (0.9~1.85)	0.168
Q4	104 (47.1)	1.85 (1.32~2.59)	<0.001	1.81 (1.29~2.55)	0.001	1.54 (1.08~2.21)	0.018
cumAIP-FI(per 1 SD)	310 (36.6)	1.35 (1.22~1.49)	<0.001	1.39 (1.25~1.54)	<0.001	1.32 (1.18~1.48)	<0.001
cumAIP-FI quartile							
Q1	51 (23.8)	1(Ref)		1(Ref)		1(Ref)	
Q2	69 (33.8)	1.55 (1.04~2.31)	0.03	1.52 (1.02~2.28)	0.04	1.4 (0.93~2.11)	0.104
Q3	78 (38.4)	1.72 (1.16~2.54)	0.006	1.64 (1.11~2.42)	0.013	1.48 (0.99~2.2)	0.053
Q4	111 (50)	2.72 (1.89~3.92)	<0.001	2.67 (1.85~3.86)	<0.001	2.23 (1.52~3.29)	<0.001
**Stroke**							
AIP-FI (per 1 SD)	107 (12.6)	1.53 (1.3~1.79)	<0.001	1.53 (1.3~1.81)	<0.001	1.35 (1.12~1.63)	0.002
AIP-FI quartile							
Q1	18 (8.7)	1(Ref)		1(Ref)		1(Ref)	
Q2	19 (8.5)	1.02 (0.51~2.05)	0.953	1 (0.5~2.03)	0.996	1.04 (0.51~2.13)	0.905
Q3	26 (13.3)	1.56 (0.8~3.05)	0.195	1.44 (0.73~2.84)	0.292	1.36 (0.68~2.68)	0.383
Q4	44 (19.9)	3.21 (1.77~5.82)	<0.001	3.14 (1.72~5.74)	<0.001	2.27 (1.2~4.3)	0.012
cumAIP-FI(per 1 SD)	107 (12.6)	1.5 (1.29~1.75)	<0.001	1.53 (1.3~1.79)	<0.001	1.38 (1.15~1.66)	0.001
cumAIP-FI quartile							
Q1	11 (5.1)	1(Ref)		1(Ref)		1(Ref)	
Q2	16 (7.8)	1.53 (0.68~3.44)	0.308	1.46 (0.64~3.3)	0.364	1.35 (0.59~3.06)	0.478
Q3	34 (16.7)	3.33 (1.62~6.84)	0.001	3.15 (1.53~6.49)	0.002	2.62 (1.26~5.44)	0.01
Q4	46 (20.7)	4.61 (2.3~9.24)	<0.001	4.5 (2.24~9.06)	<0.001	3.02 (1.45~6.27)	0.003
**DM**							
AIP-FI (per 1 SD)	268 (31.7)	1.43 (1.28~1.59)	<0.001	1.42 (1.27~1.58)	<0.001	1.33 (1.18~1.5)	<0.001
AIP-FI quartile							
Q1	37 (18)	1(Ref)		1(Ref)		1(Ref)	
Q2	64 (28.6)	1.67 (1.07~2.61)	0.024	1.75 (1.12~2.74)	0.015	1.65 (1.05~2.6)	0.031
Q3	58 (29.7)	1.91 (1.21~3.02)	0.005	1.92 (1.21~3.04)	0.006	1.76 (1.11~2.8)	0.016

Q4	109 (49.3)	3.98 (2.64~6)	<0.001	4.06 (2.68~6.14)	<0.001	3.34 (2.17~5.16)	<0.001
cumAIP-FI(per 1 SD)	268 (31.7)	1.46 (1.32~1.61)	<0.001	1.46 (1.32~1.61)	<0.001	1.38 (1.24~1.55)	<0.001
cumAIP-FI quartile							
Q1	37 (17.3)	1(Ref)		1(Ref)		1(Ref)	
Q2	46 (22.5)	1.43 (0.87~2.34)	0.157	1.45 (0.88~2.38)	0.141	1.3 (0.79~2.15)	0.305
Q3	76 (37.4)	2.92 (1.88~4.53)	<0.001	2.91 (1.87~4.52)	<0.001	2.63 (1.69~4.11)	<0.001
Q4	108 (48.6)	4.09 (2.68~6.25)	<0.001	4.09 (2.67~6.26)	<0.001	3.32 (2.13~5.18)	<0.001

Model 1 adjusted for age, sex; Model 2 adjusted for variables in Model 1 and residence, educational level, marital status, smoking status, and drinking status; Model 3 adjusted for variables in Model 2 and sleep duration, physical activity, BMI, history of hypertension and dyslipdemia, LDL-C and UA.

HR - hazard ratio, CI - confidence interval, Ref - reference, LDL-C - low-density lipoprotein cholesterol, UA - uric acid

Table S15 E-values for the association between AIP-FI and new-onset incident CMM.

Disease	Viariable	E-Value	E-Value for Lower limit of 95%CI
CMM	AIP-FI	1.95	1.76
	Cululative AIP-FI	2.28	2.10
Heart disease	AIP-FI	1.49	1.34
	Cululative AIP-FI	1.67	1.51
Stroke	AIP-FI	1.74	1.54
	Cululative AIP-FI	2.04	1.86
DM	AIP-FI	1.69	1.54
	Cululative AIP-FI	1.90	1.76

E-values were calculated based on hazard ratios from the fully adjusted model (Model 3).

CMM - cardiometabolic multimorbidity, DM - diabetes mellitus, AIP-FI - atherogenic index of plasma-frailty index

Table S16 Schoenfeld Residuals Test for Proportional Hazards Assumption.

Outcome	Exposure	N	Events	Global χ^2	df	Global p-value
CMM	Baseline AIP-FI (per 1 SD)	4157	612	11.7414	15	0.6985
CMM	Cumulative AIP-FI (per 1 SD)	4157	612	12.0559	15	0.6748
CMM	K-means cluster (2 vs 1)	4157	612	15.901	15	0.3887
Heart disease	Baseline AIP-FI (per 1 SD)	4157	1398	35.864	15	0.0018
Heart disease	Cumulative AIP-FI (per 1 SD)	4157	1398	35.3304	15	0.0022
Heart disease	K-means cluster (2 vs 1)	4157	1398	38.4397	15	0.0008
Stroke	Baseline AIP-FI (per 1 SD)	4157	534	23.0894	15	0.0823
Stroke	Cumulative AIP-FI (per 1 SD)	4157	534	25.0144	15	0.0498
Stroke	K-means cluster (2 vs 1)	4157	534	26.0817	15	0.0372
DM	Baseline AIP-FI (per 1 SD)	4157	1244	36.4742	15	0.0015
DM	Cumulative AIP-FI (per 1 SD)	4157	1244	38.0857	15	0.0009
DM	K-means cluster (2 vs 1)	4157	1244	35.2743	15	0.0022

Models were adjusted for age, sex, marriage status, residence, education, smoking status, drinking status, sleep duration, physical activity, BMI, history of hypertension and dyslipdemia, LDL-C and UA. Global test $p < 0.05$, but the exposure variable of interest satisfied the PH assumption ($p > 0.05$ for the exposure variable). The global model violation was attributable to covariates (primarily age), not the primary exposure. All exposure variables in all models had $p > 0.05$ for the Schoenfeld test.

AIP - the atherogenic index of plasma, FI - frailty index, cumAIP-FI - cumulative atherogenic index of plasma-frailty index, CMM -cardiometabolic multimorbidity, DM - diabetes mellitus

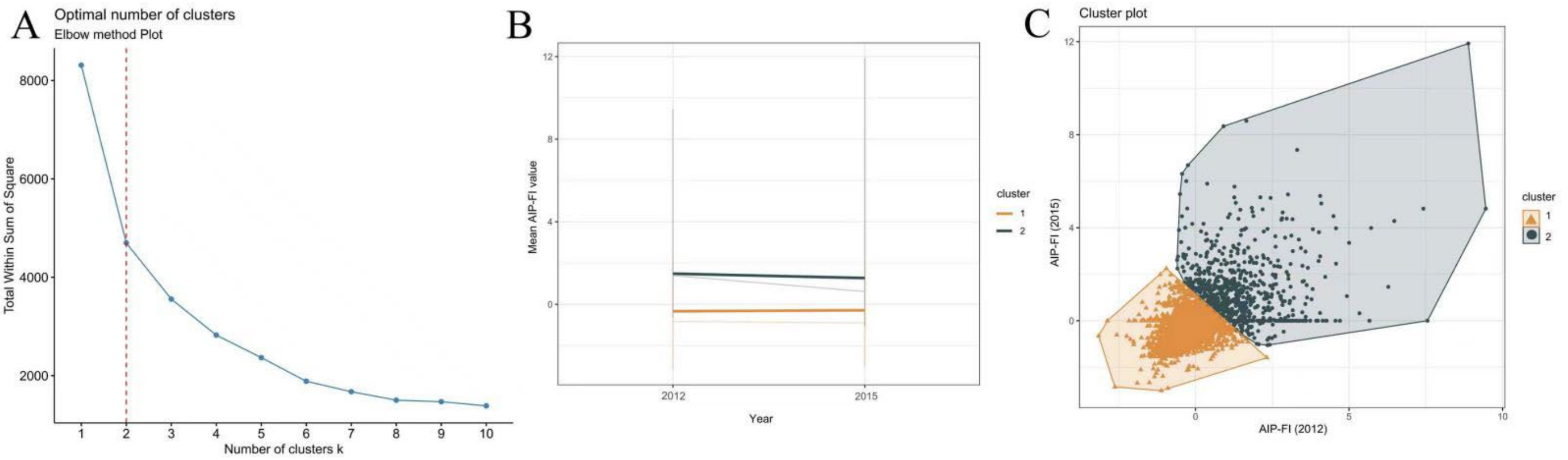

Figure S1. Clustering of the change in the AIP-FI from 2012-2015. **Panel A.** Elbow method to determine the optimal number of clusters based on within-cluster sum of squares. **Panel B.** Parallel Coordinate Plot of Mean AIP-IF Trajectories for Two Identified Clusters (2012-2015). **Panel C.** Two clusters identified by K-means analysis using Euclidean distance are visualized in a two-dimensional principal component space, demonstrating their separation.

AIP -the atherogenic index of plasma, FI - frailty index

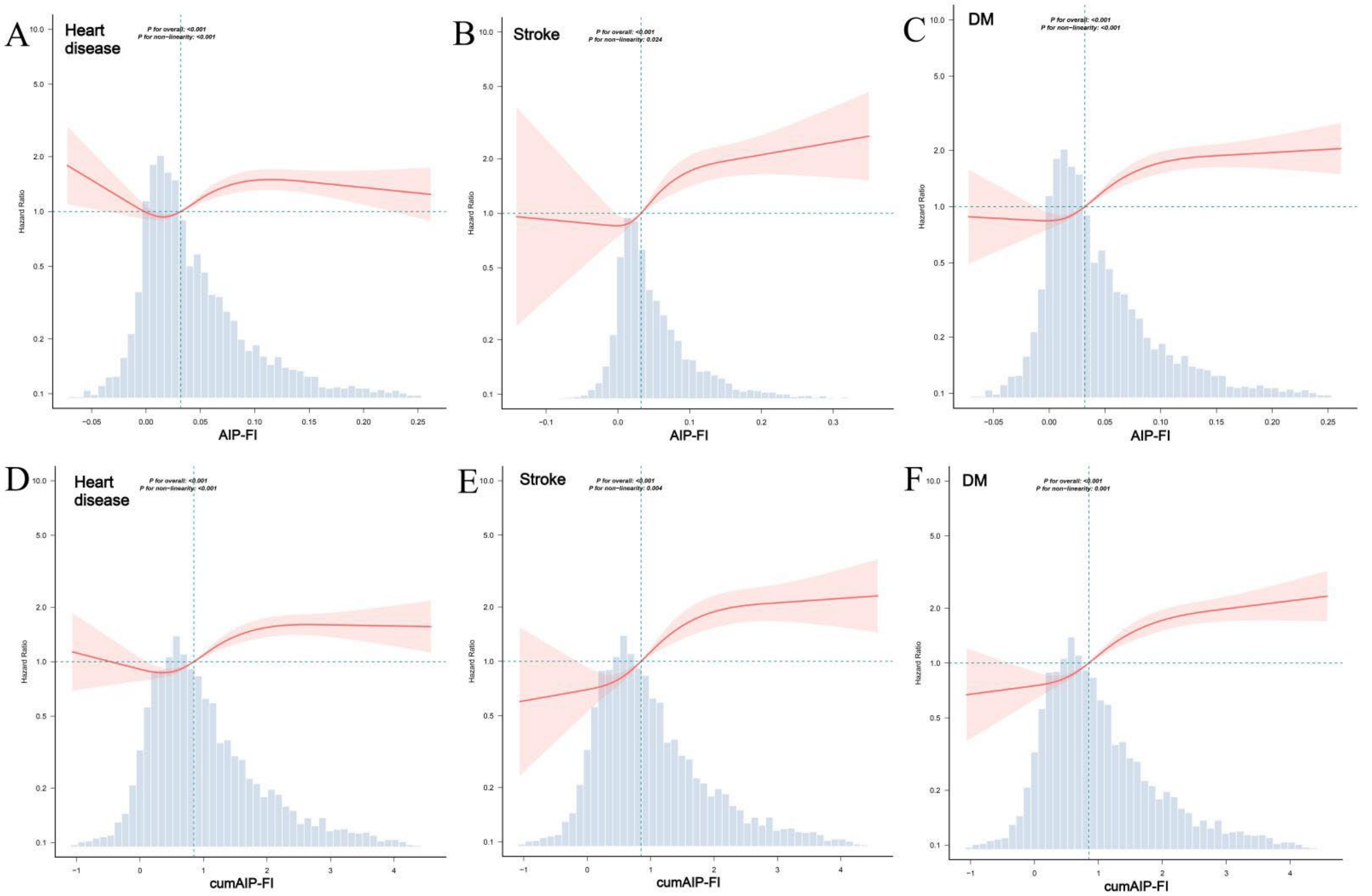

Figure S2 Restricted cubic spline curves for CMDs after covariate adjustment. **Panel A.** Restricted cubic spline curves for heart disease by AIP-FI. **Panel B.** Restricted cubic spline curves for stroke by AIP-FI. **Panel C.** Restricted cubic spline curves for DM by AIP-FI. **Panel D.** Restricted cubic spline curves for heart disease by cumulative AIP-FI. **Panel E.** Restricted cubic spline curves for stroke by cumulative AIP-FI. **Panel F.** Restricted cubic spline curves for DM by cumulative AIP-FI.

Heavy central line represents the estimated adjusted hazard ratio, with shaded ribbons denoting 99.8% confidence interval. The model is adjusted for age, sex, residence, educational level, marital status, smoking status, and drinking status, sleep duration, physical activity, BMI, history of hypertension and dyslipdemia, LDL-C and UA.

CMDs - cardiovascular metabolic diseases, AIP - the atherogenic index of plasma, FI - frailty index, cumAIP-FI - cumulative atherogenic index of plasma-frailty index, DM - diabetes mellitus

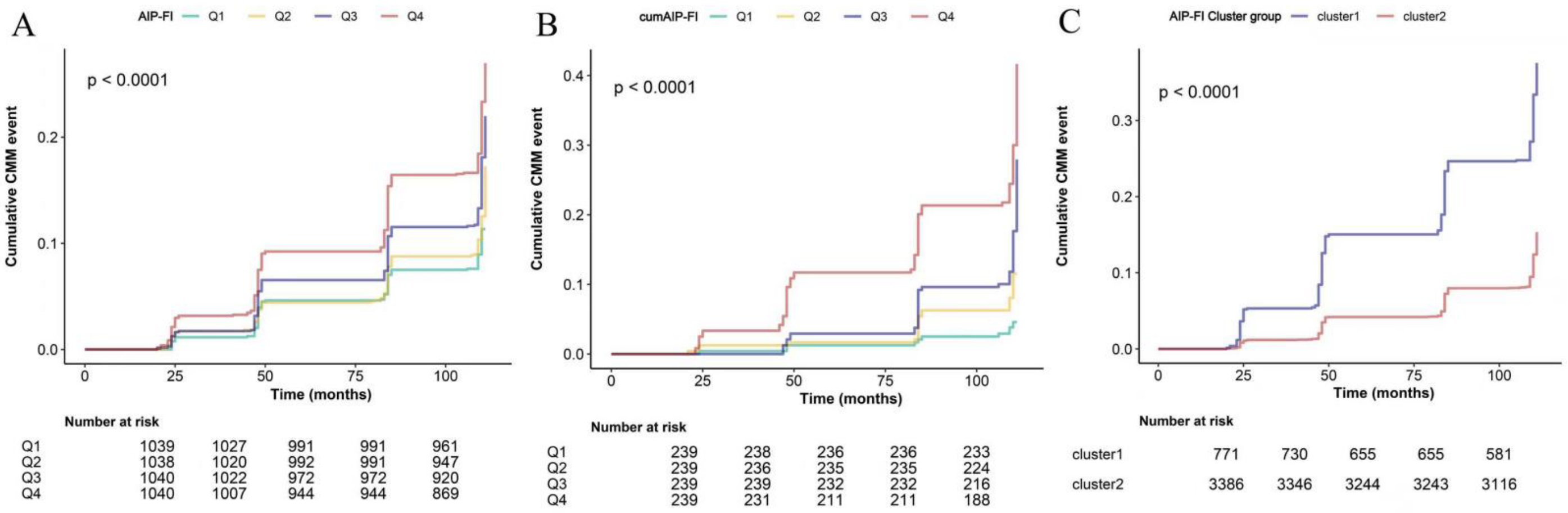

Figure S3 Kaplan-Meier curves of cumulative incidence of CMM based on baseline, cumulative, longitudinal clustering AIP-FI groupings. **Panel A.** Cumulative incidence curves for CMM by baseline AIP-FI group. **Panel B.** cumulative incidence curves for CMM by Cumulative AIP-FI group. **Panel C.** cumulative incidence curves for CMM by AIP-FI cluster group.

AIP - the atherogenic index of plasma, FI - frailty index, cumAIP-FI - cumulative atherogenic index of plasma-frailty index, CMM - cardiometabolic multimorbidity, DM - diabetes mellitus

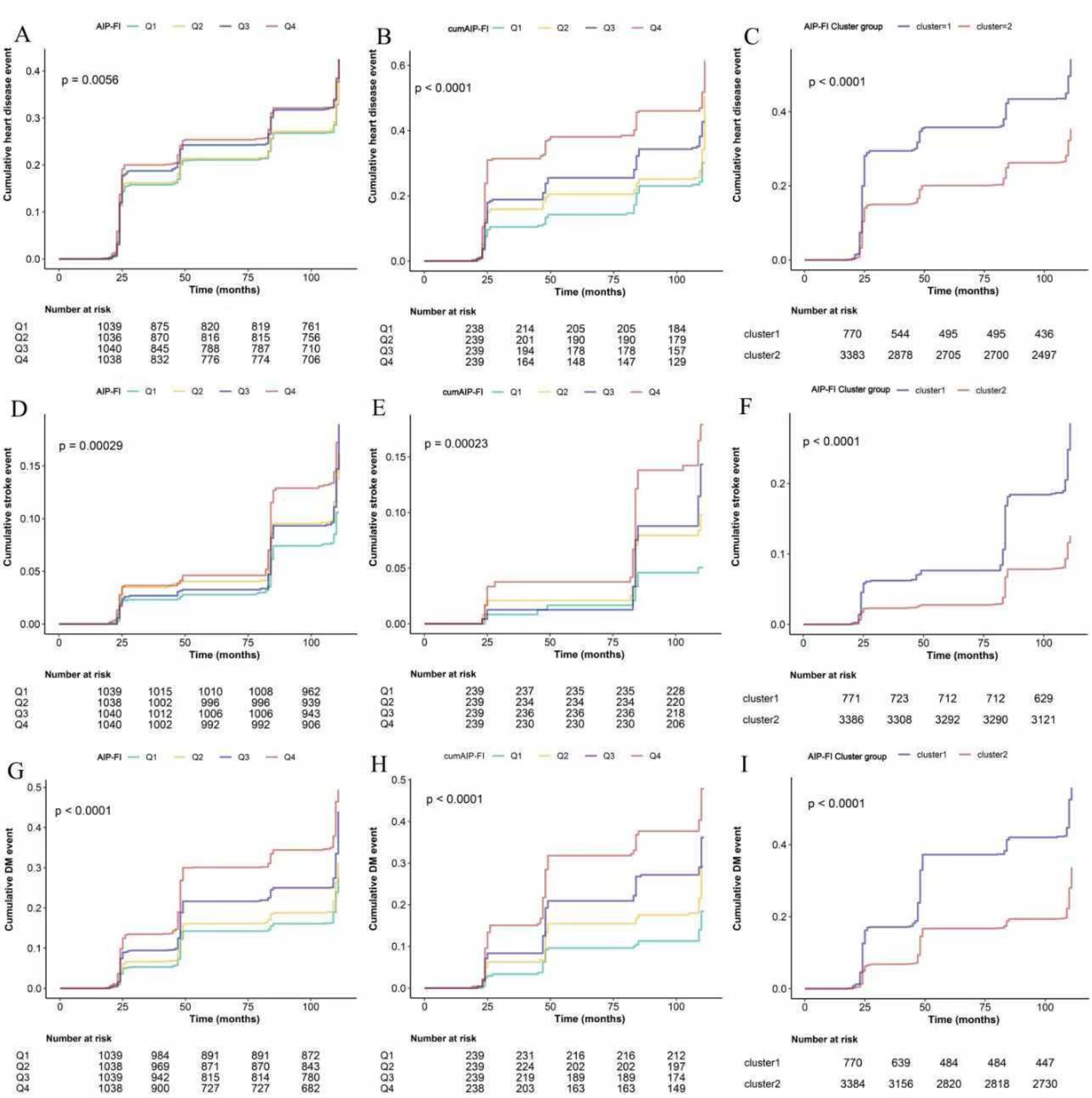

Figure S4 Kaplan-Meier curves of cumulative incidence of heart disease, stroke, and DM based on baseline, cumulative, longitudinal clustering AIP-FI groupings. **Panel A.** Cumulative incidence curves for heart disease by baseline AIP-FI groupings. **Panel B.** Cumulative incidence curves for heart disease by cumulative AIP-FI groupings. **Panel C.** Cumulative incidence curves for heart disease by longitudinal clustering AIP-FI groupings. **Panel D.** Cumulative incidence curves for stroke by baseline AIP-FI groupings. **Panel E.** Cumulative incidence curves for stroke by cumulativeAIP-FI groupings. **Panel F.** Cumulative incidence curves for stroke by longitudinal clustering AIP-FI groupings. **Panel G.** Cumulative incidence curves for DM by baseline AIP-FI groupings. **Panel H.** Cumulative incidence curves for DM by cumulative AIP-FI groupings. **Panel I.** Cumulative incidence curves for DM by longitudinal clustering AIP-FI groupings.

AIP - the atherogenic index of plasma, FI - frailty index, cumAIP-FI - cumulative atherogenic index of plasma-frailty index, DM - diabetes mellitus

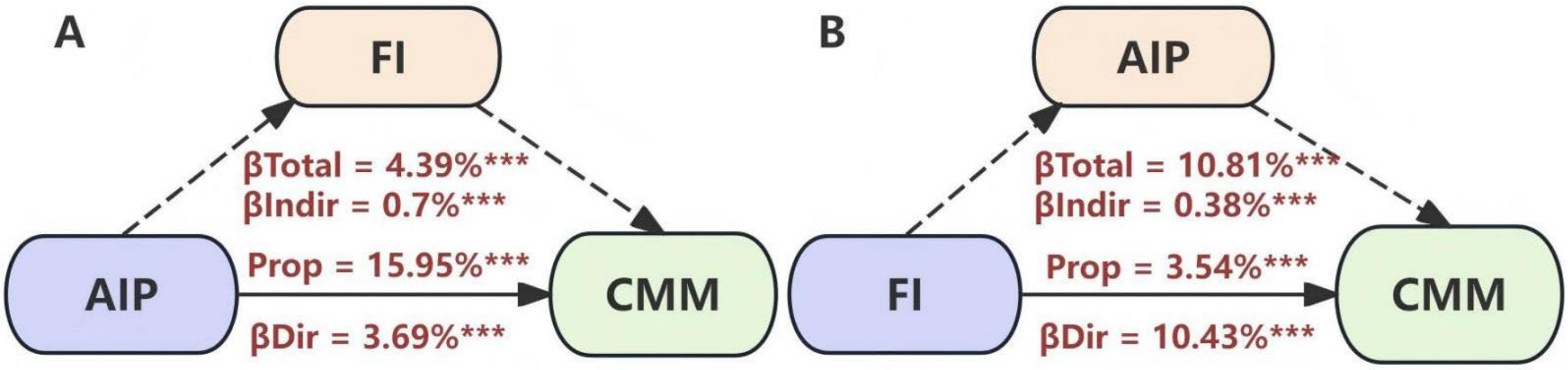

Figure S5 Mediation analysis of FI and AIP on the risk of CMM. **Panel A.** Mediation effect of FI on the association between AIP and CMM. **Panel B.** Mediation effect of AIP on the association between FI and CMM.

Models were adjusted for age, sex, marriage status, residence, education, smoking status, drinking status, sleep duration, physical activity, BMI, history of hypertension and dyslipdemia, LDL-C and UA. * $P < 0.05$, ** $P < 0.01$, *** $P < 0.001$.

CMM - cardiometabolic multimorbidity, AIP - the atherogenic index of plasma, FI - frailty index

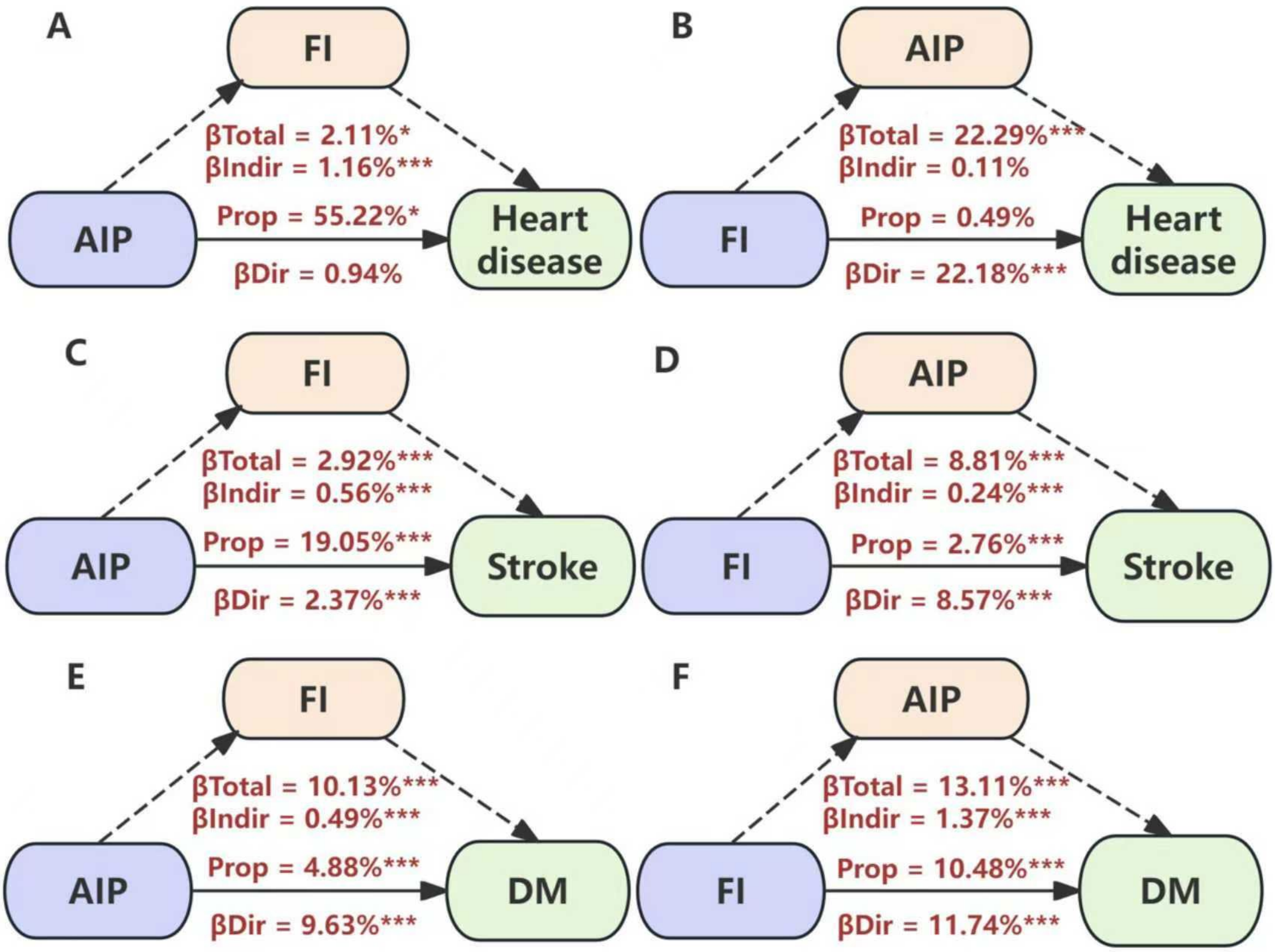

Figure S6 Mediation analysis of FI and AIP on the risk of CMDs. **Panel A.** Mediation effect of FI on the association between AIP and heart disease. **Panel B.** Mediation effect of AIP on the association between FI and heart disease. **Panel C.** Mediation effect of FI on the association between AIP and stroke. **Panel D.** Mediation effect of AIP on the association between FI and stroke. **Panel E.** Mediation effect of FI on the association between AIP and DM. **Panel F.** Mediation effect of AIP on the association between FI and DM.

Models were adjusted for age, sex, marriage status, residence, education, smoking status, drinking status, sleep duration, physical activity, BMI, history of hypertension and dyslipdemia, LDL-C and UA.* $P < 0.05$, ** $P < 0.01$, *** $P < 0.001$. CMM -cardiometabolic multimorbidity, AIP, the atherogenic index of plasma; FI, frailty index; DM, diabetes mellitus.

Subgroup	Variable	Total	Event (%)	HR (95%CI)	P for interaction
**Age, years**					
< 60	cumAIP−FI(SD)	2067	262 (12.7)	1.69 (1.53~1.86)	0.116
⩾ 60	cumAIP−FI(SD)	2090	350 (16.7)	1.59 (1.44~1.76)	
**Sex**					
Female	cumAIP−FI(SD)	2486	394 (15.8)	1.64 (1.5~1.79)	0.837
Male	cumAIP−FI(SD)	1671	218 (13)	1.62 (1.44~1.82)	
**Residence**					
Urban	cumAIP−FI(SD)	1331	208 (15.6)	1.51 (1.32~1.72)	0.315
Rural	cumAIP−FI(SD)	2826	404 (14.3)	1.72 (1.58~1.87)	
**Education level**					
Middle school below	cumAIP−FI(SD)	3164	459 (14.5)	1.64 (1.51~1.77)	0.898
Middle school and above	cumAIP−FI(SD)	993	153 (15.4)	1.68 (1.45~1.95)	
**Smoking status**					
Yes	cumAIP−FI(SD)	1132	134 (11.8)	1.94 (1.63~2.32)	0.593
No	cumAIP−FI(SD)	3025	478 (15.8)	1.63 (1.51~1.76)	
**Drinking status**					
Yes	cumAIP−FI(SD)	1201	153 (12.7)	1.86 (1.6~2.16)	0.762
No	cumAIP−FI(SD)	2956	459 (15.5)	1.63 (1.5~1.77)	
**BMI**					
< 24	cumAIP−FI(SD)	2403	246 (10.2)	1.71 (1.52~1.93)	0.126
⩾24	cumAIP−FI(SD)	1754	366 (20.9)	1.57 (1.43~1.72)	

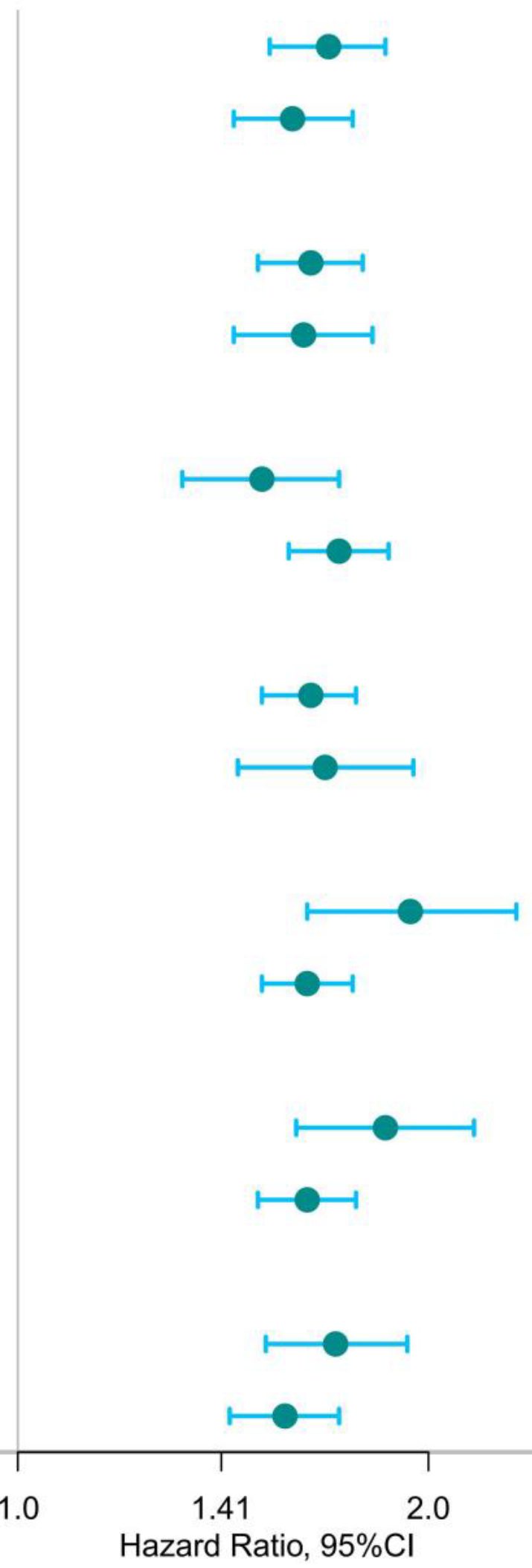

Figure S7 Subgroup analysis of the association between cumulative AIP-FI and CMM. Models were adjusted for age, sex, marriage status, residence, education, smoking status, drinking status, sleep duration, physical activity, BMI, history of hypertension and dyslipdemia, LDL-C and UA.

cumAIP-FI - cumulative atherogenic index of plasma-frailty index, HR - hazard ratio, CI - confidence interval
